# A universal wind–wave–bubble formulation for air–sea gas exchange and its impact on oxygen fluxes

**DOI:** 10.1073/pnas.2419319122

**Published:** 2025-09-16

**Authors:** Luc Deike, Xiaohui Zhou, Paridhi Rustogi, Rachel H. R. Stanley, Brandon G. Reichl, Seth M. Bushinsky, Laure Resplandy

**Affiliations:** ^a^Department of Mechanical and Aerospace Engineering, Princeton University, Princeton, NJ 08544; ^b^High Meadows Environmental Institute, Princeton University, Princeton, NJ 08544; ^c^Department of Geosciences, Princeton University, Princeton, NJ 08544; ^d^Department of Chemistry, Wellesley College, Wellesley, MA 02481; ^e^NOAA Geophysical Fluid Dynamics Laboratory, Princeton, NJ 08540; ^f^Department of Oceanography, School of Ocean and Earth Science and Technology, University of Hawai’i at Mãnoa, Honolulu, HI 96822

**Keywords:** air–sea gas exchange, bubbles, oxygen, wave breaking, supersaturation

## Abstract

Bubble-mediated gas exchange associated with wave breaking is a critical pathway for ocean–atmosphere exchange of low solubility gases such as oxygen. Yet, ocean and climate models, as well as observation-based products, usually rely on wind-only air–sea flux formulations derived from carbon constraints that ignore the asymmetric nature of the bubble flux, contributing to discrepancies between estimates of oxygen inventories and their response to climate change. Without bubbles, gas exchange is controlled by a symmetric wind-driven exchange, with the ocean–atmosphere gas partial pressure difference controlling whether outgassing or uptake occurs. Bubbles entrained by wave breaking can enhance this symmetric turbulent exchange, and contribute an additional asymmetric flux, always leading to an uptake, as they get squeezed by hydrostatic pressure (large bubbles) or collapse and fully dissolve (small bubbles). We present an observation-constrained theoretical framework of the air–sea flux accounting for air entrainment due to wave breaking and symmetric and asymmetric bubble exchange. The combined evidence from theory, laboratory, and field measurements of carbon dioxide fluxes, oxygen concentration, and noble gas supersaturation yields a universal formulation of gas exchange which we implement into a global ocean biogeochemical model. We discuss the resulting oxygen fluxes and demonstrate that our wind–wave–bubble formulation better reproduces observed in situ oxygen concentrations in water mass formation regions, where air–sea exchange is high, than a commonly used wind-only formulation. We show that the asymmetric bubble flux is essential for evaluating air–sea oxygen fluxes and estimating the magnitude of the ocean oxygen loss associated with global warming.

## Significance

Bubbles entrained by breaking waves at the ocean surface and squeezed during their underwater journey provide a critical pathway for exchange of low solubility gases such as oxygen, with profound implications for biogeochemical cycles. Ocean and climate models, as well as observation-based products, usually ignore the asymmetric contribution to air–sea gas transfer due to bubbles, leading to biases in oxygen representation. We present and implement a bubble gas exchange theory constrained by noble gas supersaturation laboratory measurements and float oxygen concentrations in wintertime in the Southern Ocean. We demonstrate an improved representation of oxygen undersaturation in a global ocean circulation model, with significant implications for our ability to predict changes in ocean oxygen content.

Gas exchange at the ocean–atmosphere interface is essential for understanding ocean biogeochemical cycles and air–sea fluxes, critical for the Earth’s climate, including the magnitude of the ocean carbon sink (1, 2) and the warming-driven ocean oxygen loss (e.g., ref. 3) associated with anthropogenic activities. Processes controlling ocean–atmosphere gas exchange involve a wide range of scales from micrometer scale bubbles, to meter scale waves, and ocean basin wide variations in wind and atmospheric pressure (4–6). Ocean and Earth system models (e.g., refs. 7–9), as well as observation-based products (e.g., refs. 10 and 11) usually represent the ocean–atmosphere gas flux F, as a function of the gas partial pressure difference between air and water ($P_{a}-P_{w}$, a measure of the disequilibrium across the interface), the gas solubility (S, the amount of gas that can dissolve for given thermodynamical conditions), and a gas transfer velocity $k_{w}$, often expressed as a function of wind speed and gas diffusivity (the ability of a gas to diffuse through the interface) (12–18):

[1] $$\begin{array}{c} F=k_{w}S\left(P_{a}-P_{w}\right). \end{array}$$

Such wind-only turbulent diffusive gas transfer velocity formulations of $k_{w}$ are relatively easy to implement and were successful at evaluating global scale ocean fluxes of the intermediate solubility gases such as CO_2_ for which they were originally designed (13, 14). However, these formulations only implicitly account for bubbles, and lack the effect of squeezed and fully dissolving bubbles entrained into the water column (4–6). These omitted bubble effects are first-order processes in the exchange of low solubility gases such as O_2_, N_2_ (19–23), and gases used as tracers to understand ocean ventilation [noble gases, SF_6_ (24, 25)], and contribute to current biases and uncertainties in air–sea oxygen fluxes and global ocean oxygen loss estimates (3, 7, 26–28). These wind-only formulations also exclude the direct control of wave breaking at a particular ocean location on the entrainment of bubbles. Local wave breaking is influenced by waves traveling far distances, which leads to multiple possible values of the gas transfer velocity $k_{w}$ at any given wind speed and introduces high-frequency and small-scale spatial variability (minute to days, meters to 100 km) in the air–sea flux (29–32).

**Fig. 1.**
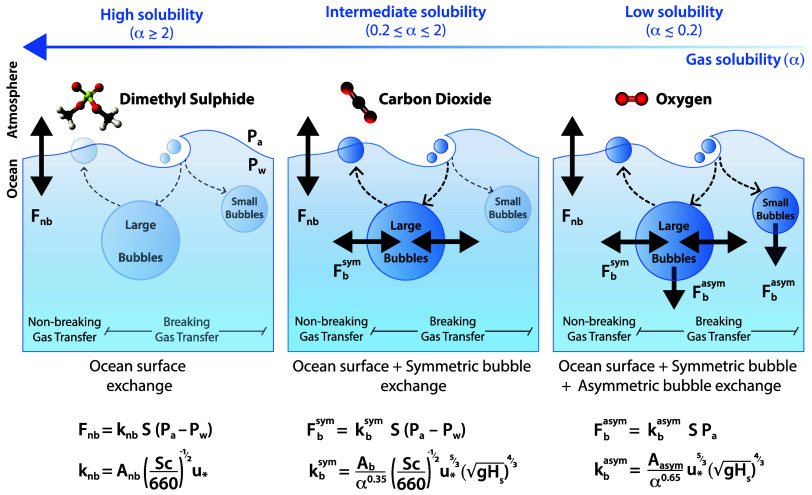
Schematic of the processes controlling gas exchange at the air–water interface for DMS (high solubility), CO_2_ (intermediate solubility) and O_2_ (low solubility), and formulations of the surface flux (F_*nb*_), the symmetric bubble flux (${\text{F}}_{b}^{\mathrm{sym}}$) and the asymmetric bubble flux (${\text{F}}_{b}^{\mathrm{asym}}$). F_*nb*_ is controlled by turbulent water surface renewal. ${\text{F}}_{b}^{\mathrm{sym}}$ is mediated by large bubbles that increase the surface area of exchange. Both are controlled by the gas partial pressure difference and are symmetric (i.e. represent either uptake or outgassing). ${\text{F}}_{b}^{\mathrm{asym}}$ is controlled by large bubbles under hydrostatic pressure and small bubbles that fully dissolve; it only depends on the gas partial pressure in the air and is always directed toward the ocean. The relative contributions of ${\text{F}}_{b}^{\mathrm{sym}}$ and ${\text{F}}_{b}^{\mathrm{asym}}$ increase as the gas solubility decreases. The Ostwald solubility is related to the aqueous gas solubility S by α=RTS with R the ideal gas constant and T the water temperature. Respective gas transfer velocities k_*nb*_, ${\text{k}}_{b}^{\mathrm{sym}}$, and ${\text{k}}_{b}^{\mathrm{asym}}$ are expressed as a function of the wind friction velocity $u_{*}$, the significant wave height $H_{s}$, Ostwald solubility α, Schmidt number Sc=ν/D (ratio of water kinematic viscosity and gas diffusivity), and constant prefactors ($A_{\mathrm{nb}}$, $A_{b}$, $A_{\mathrm{asym}}$) constrained by theory and observations.

The air–sea gas exchange can be viewed as the sum of three fluxes: a nonbreaking ocean surface flux, a symmetric bubble flux, and an asymmetric bubble flux (Fig. 1) (4, 5, 21, 22). The nonbreaking surface flux gas transfer velocity is controlled by the wind-induced turbulent renewal of surface water and the gas diffusivity. It is dominant in the absence of breaking waves and bubbles (usually at low wind speed) and for highly soluble gases (nondimensional Ostwald solubility α≳2), such as dimethylsulfide (DMS). The symmetric bubble flux is largely mediated by large bubbles that act to increase the surface area available for gas exchange as they are entrained downward and rise back up to the surface. This flux becomes important for moderately soluble gases such as CO_2_ (0.2≲α≲2) at high wind speeds and for low solubility gases such as O_2_ (α≲0.2) even at moderate wind speeds. Both the nonbreaking surface flux and the symmetric bubble flux can lead to either uptake or outgassing (flux sign depends on the partial pressure difference between air and water). Finally, the asymmetric bubble flux occurs when large bubbles get squeezed by hydrostatic pressure during their journey in the water column or when small bubbles get crushed and fully dissolve (4, 5, 21, 22). In contrast to the nonbreaking and symmetric bubble fluxes, the asymmetric bubble flux only depends on the air partial pressure, systematically leads to gas uptake by the ocean, and can induce supersaturation in the water column (5, 24, 33, 34). The magnitude of this asymmetric bubble flux increases as solubility decreases and becomes a major factor for low solubility gases such as O_2_. The mechanistic influence of the asymmetric bubble-mediated flux is supported by in situ observations that show noble gas and oxygen supersaturations of 1 to 2% (21, 24, 33). During winter storms with high wave activity, the asymmetric bubble flux could even change the sign of the air–sea flux, where outgassing would be expected from the gas partial pressure difference, but where uptake could actually occur (23, 35–37).

In the present paper, we propose a formulation of the symmetric and asymmetric bubble-mediated fluxes (large and small bubbles) which is valid for gases across a wide range of solubility that play important roles in the Earth’s climate system (CO_2_, O_2_, N_2_, N_2_O, noble gases, SF_6_). The formulation is grounded theoretically (4–6, 30), and carefully constrained by multiple empirical observations spanning a wide range of gases and meteorological conditions, including fluxes of DMS and CO_2_ in the open ocean (18, 29, 38, 39), laboratory and field data on noble gas supersaturation (33, 34) and recent float data on oxygen concentration in the Southern Ocean (40, 41). Previous attempts to parameterize bubble-mediated gas exchange have largely been fitted from limited datasets acquired at specific locations and specific gases (19–22, 33), and therefore often fail when used globally or when extrapolating to other gases. Our observation-constrained theoretical formulation accounts for wind, waves, and bubbles, with wave breaking modulating the gas exchange at a given wind speed (18, 30–32), and the dependence on solubility and diffusivity of the gases, with much stronger bubble-mediated gas exchange for low solubility gases (Fig. 1). Finally, we implement the proposed formulation in a global ocean circulation model and discuss implications for global ocean oxygen loss.

The paper is organized as follows: We present the theory, validate the bubble contribution against noble gas supersaturation laboratory experiments at high wind speed, derive a simple wind–wave–bubble formulation, and finally implement it in a global ocean model to discuss implications for the ocean oxygen fluxes.

## Gas Flux Theory Accounting for Wind, Waves, and Bubbles

We first present the gas flux theory developed by Keeling (5) and Deike and Melville (30).

### Fluxes.

We separate the gas exchange as the sum of the nonbreaking flux through the ocean surface $F_{\mathrm{nb}}$ and through bubbles, itself separated into a symmetric bubble flux $F_{b}^{\mathrm{sym}}$ and an asymmetric bubble flux $F_{b}^{\mathrm{asym}}$ (4, 5)

[2] $$\begin{array}{c} F=F_{\mathrm{nb}}+F_{b}^{\mathrm{sym}}+F_{b}^{\mathrm{asym}}. \end{array}$$

The surface and bubble-symmetric fluxes are related to the partial pressure differences between the air and water side $P_{a}-P_{w}$, the gas solubility S, and a transfer velocity coefficient,

[3] $$\begin{array}{c} F_{\mathrm{nb}}=k_{\mathrm{nb}}S\left(P_{a}-P_{w}\right), \end{array}$$

[4] $$\begin{array}{c} F_{b}^{\mathrm{sym}}=k_{b}^{\mathrm{sym}}S\left(P_{a}-P_{w}\right). \end{array}$$

The sign of the flux is driven by the difference in partial pressure between the air side and the water side (higher partial pressure in water leads to an outgassing or evasion and a negative flux, while higher partial pressure in the air leads to uptake or invasion and a positive flux). The surface gas transfer velocity $k_{\mathrm{nb}}$ depends on wind speed and gas diffusivity and will be dominant for highly soluble gases such as DMS. The symmetric bubble transfer velocity $k_{b}^{\mathrm{sym}}$ is controlled by large bubbles getting into equilibrium as they spend a finite time under water. The symmetric (large) bubble transfer contribution is important to understand CO_2_ gas transfer velocity at high wind speed (30, 32), and its importance increases as the gas solubility decreases (5, 30).

The asymmetric bubble term, important for gases with low solubility such as O_2_, accounts for the bubbles that do not equilibrate while they are under water and only transfer gas into the water as they get squeezed or crushed by hydrostatic pressure. The functional form of this asymmetric flux is

[5] $$\begin{array}{c} F_{b}^{\mathrm{asym}}=k_{b}^{\mathrm{asym}}SP_{a}, \end{array}$$

where $k_{b}^{\mathrm{asym}}\geq 0$. The asymmetric flux scales with the atmospheric partial pressure of the gas considered and will systematically drive gas uptake into the water.

### Nonbreaking Ocean Surface Exchange.

The ocean surface flux $F_{\mathrm{nb}}$ is driven by diffusive mass transfer at the air–sea interface, enhanced by turbulence, and is given by Eq. **3**. Following eddy-renewal theory, $k_{\mathrm{nb}}$ scales with the Schmidt number (Sc=ν/D the ratio of water kinematic viscosity and gas diffusivity in water) as $Sc^{-1/2}$, and linearly with the friction velocity $u_{*}$, which is related to the wind speed at 10 m but considers the role of atmospheric stability, relative ocean surface current velocity, and is a more direct driver of the upper ocean turbulence and the air–sea fluxes (42–44),

[6] $$\begin{array}{c} k_{\mathrm{nb}}=A_{\mathrm{nb}}u_{*}{\left(Sc/660\right)}^{-1/2}. \end{array}$$

The coefficient $A_{\mathrm{nb}}$ is a nondimensional prefactor that was previously constrained by field observations of DMS and CO_2_ fluxes, with an estimated 20% uncertainty (29, 30, 32, 38, 45).

### Symmetric Bubbles Exchange.

The symmetric bubble flux (Eq. **4**) accounts for large bubbles with a finite residence time, with the transfer coefficient given in refs. 5 and 30,

[7] $$\begin{array}{c} k_{b}^{\mathrm{sym}}=\frac{V_{\mathrm{exch}}}{\alpha }=\frac{1}{\alpha }\int_{R_{\mathrm{inj}}}^{R_{\max}}dR_{b}\left(4\pi /3\right)R_{b}^{3}Q\left(R_{b}\right)E\left(R_{b}\right), \end{array}$$

where α=RTS is the Ostwald solubility (R the ideal gas constant and T the water temperature), $V_{\mathrm{exch}}$ is the gas volume per unit time per unit ocean surface area being exchanged, $E(R_{b})$ is an efficiency factor dependent on the bubble size $R_{b}$, $Q(R_{b})$ is the bubble distribution flux across the air–sea interface, $R_{\max}$ is the maximum bubble size and $R_{\mathrm{inj}}$ the small bubble cut-off size. The efficiency factor is controlled by the depth of bubble injection $z_{0}$, and an equilibration depth, itself a function of the individual bubble gas exchange, rise velocity, and the gas solubility and diffusivity (see details in *Materials and Methods*, *SI Appendix*, and refs. 5 and 30).

### Asymmetric Bubbles Exchange.

The asymmetric gas transfer velocity used in the asymmetric flux Eq. **5** is derived in ref. 5

[8] $$\begin{array}{c} k_{b}^{\mathrm{asym}}=\frac{V_{\mathrm{inj}}}{\alpha }+\frac{\Delta P}{P_{0}}\frac{V_{\mathrm{exch}}}{\alpha }, \end{array}$$

and includes the contribution of small fully dissolving bubbles, $V_{\mathrm{inj}}$, and of larger bubbles getting squeezed by hydrostatic pressure $\Delta P/P_{0}$ (5), a term which depends on the bubble injection depth, rise velocity, and individual exchange coefficient, both being evaluated theoretically; see *Materials and Methods* and *SI Appendix*. The injected volume is $V_{\mathrm{inj}}=\int_{R_{\min}}^{R_{\mathrm{inj}}}Q\left(R_{b}\right)4/3\pi R_{b}^{3}dR_{b}$, with $R_{\min}$ the smallest bubbles considered. The size cut-off $R_{\mathrm{inj}}$ for the small injected bubbles contained in $V_{\mathrm{inj}}$ is estimated between 50 to 300 μm (6), corresponding to bubbles with rise velocities smaller than the water turbulence fluctuations, hence bubbles unable to rise back to the surface and that will eventually collapse due to hydrostatic and surface tension pressure.

## Validation of the Bubble Gas Flux Theory Against Noble Gas Laboratory Experiments at High Wind Speed

The bubble gas flux theory (Eqs. **7** and **8**) was previously lacking adequate measurements to constrain the dependency on solubility across gases. Here, we use the laboratory measurements from ref. 34 recently performed at the Miami SUSTAIN facility in a high wind speed regime (wind speed equivalent at 10 m up to 50 m s^−1^, for various forced wave conditions) (46, 47). These measurements showed that the noble gas supersaturation increased for winds up to 35 m s^−1^ and then leveled off at higher wind speed, suggesting an asymptotic bubble dominated regime. In this saturated regime, we can expect that the gas exchange is dominated by bubbles, providing an ideal framework to validate and constrain the gas flux bubble theory across a wide range of solubility. We focus our analysis on the set of measurements above the asymptotic high wind speed limit and use these data to validate our bubble gas exchange theory, specifically the dependency of the gas transfer velocity $k_{b}^{\mathrm{sym}}$ and $k_{b}^{\mathrm{asym}}$ with solubility together with the associated predicted supersaturation.

**Fig. 2.**
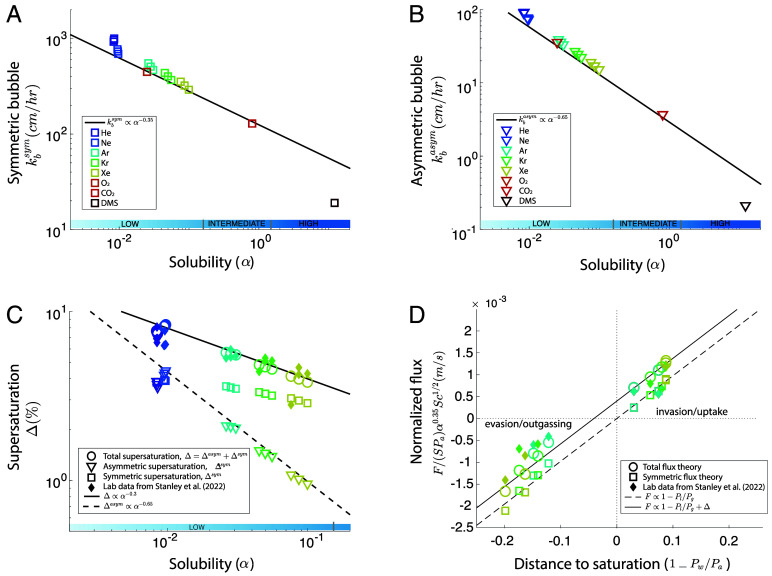
Gas flux theory at the high wind speed limit in laboratory conditions ($U_{10}>40$ m s^−1^) from ref. 34. (*A*) Symmetric bubble contribution $k_{b}^{\mathrm{sym}}$ and b) Asymmetric bubble contribution in cm hr^−1^ as a function of solubility α for multiple gases. The gas flux theory (symbols) suggests $k_{b}^{\mathrm{sym}}\propto \alpha^{-0.35}$ (*A*) and $k_{b}^{\mathrm{asym}}\propto \alpha^{-0.65}$ (*B*) shown in black lines, for α≲2. Note that the symmetric term has a diffusivity dependency. (*C* and *D*) Supersaturation and gas exchange in the asymptotic regime of high wind speed comparing noble gases laboratory data (34) and our gas flux theory [He, Ne, Ar, Kr, Xe color-coded as in (*A* and *B*)]. (*C*) Supersaturation. Quantitative agreement between the laboratory data [solid diamonds (34)] and theoretical total supersaturation is observed (open circles), summing the asymmetric contribution (open triangles) and the symmetric contribution (open squares). Solid and dashed lines indicate power laws $\alpha^{-0.65}$ (for the asymmetric contribution) and $\alpha^{-0.3}$ (for the total contribution). Recall that the total supersaturation can be related in the theory to the exchange coefficient as $\Delta =k_{b}^{\mathrm{asym}}/\left(k_{b}^{\mathrm{sym}}+k_{\mathrm{nb}}\right)\approx k_{b}^{\mathrm{asym}}/k_{b}^{\mathrm{sym}}\propto \alpha^{-0.3}$ which is observed in the data and theory. (*D*) Normalized gas flux $F\alpha^{0.35}Sc^{1/2}/\left(SP_{a}\right)$ as a function of the distance to saturation $1-P_{w}/P_{a}$. Positive values of the distance to saturation indicate invasion/uptake, while negative values indicate evasion/outgassing. Solid diamonds are laboratory data (34). Open circles indicate the total theoretical flux $F=F_{\mathrm{sym}}+F_{\mathrm{asym}}$ which describes the data well and is systematically shifted so that invasion fluxes are larger than evasion for the same distance to saturation $1-P_{w}/P_{a}$ (solid line). Open squares indicate the modeled symmetric flux $F_{\mathrm{sym}}=\left(k_{\mathrm{nb}}+k_{b}^{\mathrm{sym}}\right)S\Delta p$. The theoretical symmetric flux lies on a linear scaling going through the origin (dashed line). Notably, all experimental data are above the theoretical symmetric flux indicating the need for the asymmetric bubble flux contribution $F_{\mathrm{asym}}=k_{b}^{\mathrm{asym}}SP_{a}$.

We calculate the symmetric and asymmetric transfer velocity from Eqs. **7** and **8** with realistic bubble size distribution, rise velocity, and individual bubble gas exchange, an injected depth of $z_{0}=0.5$ m, and $R_{\mathrm{inj}}=150\;\mu$m. (see *Materials and Methods* and refs. 30 and 32), all consistent with the high wind speed laboratory conditions of ref. 34. Fig. 2 *A* and *B* show the symmetric bubbles (Eq. **7**, panel *A*) and the asymmetric bubbles (Eq. **8**, panel *B*) gas transfer velocity for noble gases, CO_2_, O_2_, and DMS, spanning a wide range of solubility. We find that the bubble theory for various gases (symbols) can be described by a scaling (solid line) for the symmetric term, by $k_{b}^{\mathrm{sym}}\propto Sc^{-1/2}\alpha^{-0.35}$ (Fig. 2*A*) and for the asymmetric small bubble, by $k_{b}^{\mathrm{asym}}\propto \alpha^{-0.65}$ (Fig. 2*B*), with a very weak dependence in diffusivity. We note the power laws are valid for solubility below a threshold, α≲2, with the bubble contribution dropping dramatically for highly soluble gases (such as DMS).

Further, we observe that all experimental data of noble gas supersaturation Δ (solid diamonds for He, Ne, Ar, Kr, Xe) can be reasonably described by a simple scaling $\Delta \propto \alpha^{-0.3}$ while diffusivity only plays a minor role (Fig. 2*C*). This observation can be explained and predicted by the theoretical bubble induced supersaturation, calculated as the excess concentration in the water due to the bubbles (see *Materials and Methods* and ref. 5). Fig. 2*C* shows that the total theoretical bubble induced supersaturation describes quantitatively the experimental data (open circles are nearly on top of the diamonds). The supersaturation arises from both the large (compressed by hydrostatic pressure) and small (fully dissolved) bubbles (5). Small fully dissolving bubbles have a strong supersaturation scaling with α and a weak scaling in diffusivity (open triangles), while large squeezed bubbles have a weak scaling in α and stronger scaling in diffusivity (open squares). The sum of both contributions (open circles) reproduces the scaling and magnitude of the laboratory data.

The ability to predict theoretically the asymptotic (high wind speed) supersaturation across a wide range of solubility is an important validation, and highlights the importance of both large and small bubbles. The scaling $\Delta \propto \alpha^{-0.3}$ can be understood since, in the asymptotic high wind speed regime, with the gas exchange dominated by bubbles, we have $k_{\mathrm{nb}}\ll k_{b}^{\mathrm{sym}}$, so that $\Delta \propto k_{b}^{\mathrm{asym}}/k_{b}^{\mathrm{sym}}\propto \alpha^{-0.3}$. Note that previous formulations including bubble effects (20, 21, 33) would not lead to the observed supersaturation scaling $\Delta \propto \alpha^{-0.3}$ (*SI Appendix*, Fig. S4), and do not have a dependency in solubility in their symmetric transfer velocity, while the wind-only formulation Eq. **1** (17) would not predict any supersaturation.

Having successfully compared the bubble model against supersaturation obtained in the laboratory, we now consider the associated fluxes estimated by measuring the partial pressure in the air as well as the dissolved gas concentration in the water over time by ref. 34. Their experiments were designed to induce either invasion or evasion (by controlling the water temperature) and demonstrated that the invasion flux was systematically larger than the evasion flux due to the asymmetric role of the bubbles (34).

Our bubble theory predicts the gas transfer coefficients, and we use the solubility, partial pressure in the air $P_{a}$ and water $P_{w}$ measured in the experiments to obtain the fluxes, which can then be compared to the measured fluxes. We can rewrite the flux (neglecting the surface contribution) as $F/\left(k_{b}^{\mathrm{sym}}SP_{a}\right)\propto F/SP_{a}\alpha^{0.35}Sc^{1/2}\propto 1-P_{w}/P_{a}+\Delta$; to compare fluxes for gases with very different partial pressure (and solubility) and highlight the differences between evasion and invasion.

Fig. 2*D* shows both invasion and evasion fluxes, following the proposed normalization, i.e. $F/SP_{a}\alpha^{0.35}Sc^{1/2}$ as a function of the distance to saturation $1-P_{w}/P_{a}$. Fluxes obtained from the theory (open circles, summing symmetric and asymmetric contributions) match the laboratory data (solid diamonds). They also reproduce the systematically stronger invasion compared to evasion for a given distance to saturation (e.g., normalized uptake of ≈+1.2 m s^−1^ for $1-P_{w}/P_{a}=0.1$ vs. outgassing of ≈−0.6 m s^−1^ for $1-P_{w}/P_{a}=-0.1$, Fig. 2*D*). In contrast, the symmetric flux (open squares) falls on a linear scaling going through the origin as expected (same positive or negative flux for the same distance to saturation) but is not sufficient to describe the data. The asymmetric flux is necessary and the shift can be directly related to the supersaturation.

We note that the flow in the laboratory will never exactly mimic the one in the field; the scales of the waves or bubble penetration depth can be different for a similar nominal wind speed in the laboratory and in the open ocean. We use the laboratory to quantify a bubble dominated regime, obtain the scaling of noble gas supersaturation with solubility and diffusivity, and constrain the bubble contributions. Such scaling can then be combined with established theories on air entrainment as a function of wave and wind variables in the open ocean, discussed below. As such, the present modeling aims to incorporate bubbles in the near surface as well as beneath the breaking wave layer. A fundamental assumption being that the bubble size distribution is similar in the open ocean and in the laboratory. Uncertainties in the field of the total air entrainment and the penetration depth of the bubble plume remain and deserve further field investigations.

## Deriving the Wind–Wave–Bubble Formulation

We now derive a simpler wind–wave–bubble formulation based on the full gas flux theory described above.

We have obtained and validated the scaling of the symmetric and asymmetric gas transfer velocity with solubility and diffusivity (Fig. 2 *A* and *B*). The dependency with wind and waves is related to air entrainment by wave breaking (30, 48), quantified by the total volume of air entrained by breaking waves $V_{A}=\int dR_{b}\left(4\pi /3\right)R_{b}^{3}Q\left(R_{b}\right)$. As such, the magnitude of the bubble size distribution flux $Q(R_{b})$ controls the bubble exchange $V_{\mathrm{exch}}$ and injection $V_{\mathrm{inj}}$ volumes, so that $V_{\mathrm{exch}}\propto V_{A}$ and $V_{\mathrm{inj}}\propto V_{A}$. The entrained volume $V_{A}$ is a volumetric analog to the whitecap coverage, which depends on the wind and wave variables through wave breaking (6, 49, 50) and controls air–sea fluxes of gases (30, 32) and sea spray aerosols (51). The bubble flux $Q(R_{b})$ and associated scale-dependent integration for $V_{A}$ is well approximated by a simpler scaling relationship with the significant wave height $H_{s}$ and the wind friction velocity $u_{*}$ based on field measurements of the breaking statistics, leading to $V_{A}\propto u_{*}^{5/3}{\sqrt{gH_{s}}}^{4/3}$ (30, 48, 49).

Recombining the variables, we obtain the wind–wave–bubble formulation for the symmetric bubble gas transfer coefficient,

[9] $$\begin{array}{c} k_{b}^{\mathrm{sym}}=A_{b}u_{*}^{5/3}{\sqrt{gH_{s}}}^{4/3}{\left(Sc/660\right)}^{-1/2}\alpha^{-0.35}, \end{array}$$

where the coefficient $A_{b}$ has already been constrained (together with $A_{\mathrm{nb}}$) against gas transfer velocity measurements for CO_2_ (30, 32), and is summarized in Table 1.

**Table 1.** Wind–wave–bubble formulation for gas exchange: nonbreaking, symmetric, and asymmetric bubble contributions

	Wind–wave–bubble formulation		
	Nonbreaking	Symmetric bubbles	Asymmetric bubbles
Flux (mol m^−2^ s^−1^)	$F_{\mathrm{nb}}=k_{\mathrm{nb}}S\left(P_{a}-P_{w}\right)$	$F_{b}^{\mathrm{sym}}=k_{b}^{\mathrm{sym}}S\left(P_{a}-P_{w}\right)$	$F_{b}^{\mathrm{asym}}=k_{b}^{\mathrm{asym}}SP_{a}$
Transfer velocity (m s^−1^)	$k_{\mathrm{nb}}=A_{\mathrm{nb}}\frac{u_{*}}{{\left(Sc/660\right)}^{1/2}}$	$k_{b}^{\mathrm{sym}}=A_{b}\frac{u_{*}^{5/3}{\sqrt{gH_{s}}}^{4/3}}{\alpha^{0.35}{\left(Sc/660\right)}^{1/2}}$	$k_{b}^{\mathrm{asym}}=A_{\mathrm{asym}}\frac{u_{*}^{5/3}{\sqrt{gH_{s}}}^{4/3}}{\alpha^{0.65}}$
Coefficients	$A_{\mathrm{nb}}=1.33\cdot 10^{-4}$	$A_{b}=1.2\cdot 10^{-5}$ (m^−2^*s*^2^)	$A_{\mathrm{asym}}=7\cdot 10^{-8}$ (m^−2^*s*^2^)
Uncertainty	$\pm 0.1\cdot 10^{-4}$	$\pm 0.1\cdot 10^{-5}$ (m^−2^*s*^2^)	$\pm 4\cdot 10^{-8}$ (m^−2^*s*^2^)
Theory constraints	Eddy renewal	Air entrained and Bubble model	Air entrained and Bubble model
Data constraints	Field CO_2_ and DMS fluxes at low wind speed	Field CO_2_ fluxes at moderate to high wind speed	Lab and field supersaturation of noble gases

Coefficients determined using COARE momentum flux formulation (52) to link $u_{*}$ and $U_{10}$. Coefficients using NCAR momentum flux (53) and a simplified bubble formulation only retaining the wind (and not the waves) are provided in *SI Appendix*, Table S1. Note that the formulae and coefficients in the table lead to gas transfer velocity in m s^−1^, which is converted to cm hr^−1^ in the figures. Definitions of the parameters are provided in *SI Appendix*, Table S2.

Similarly, we obtain the wind–wave–bubble formulation for the asymmetric bubble transfer coefficient,

[10] $$\begin{array}{c} k_{b}^{\mathrm{asym}}=A_{\mathrm{asym}}\alpha^{-0.65}u_{*}^{5/3}{\sqrt{gH_{s}}}^{4/3}. \end{array}$$

We constrain the final parameter $A_{\mathrm{asym}}$ through the theory (integration of the full gas flux equations provides an order of magnitude constraint) and evaluate against empirical evidence. The first empirical constraint comes from field measurements of noble gas supersaturation (33), with reasonable agreement between the data and the predicted bubble induced supersaturation (*SI Appendix*, Fig. S3). The second constraint comes from the comparison of the oxygen fluxes and concentration when we implement the present formulation into a global ocean circulation model to observed values, presented below. The value of $A_{\mathrm{asym}}$ is provided in Table 1.

**Fig. 3.**
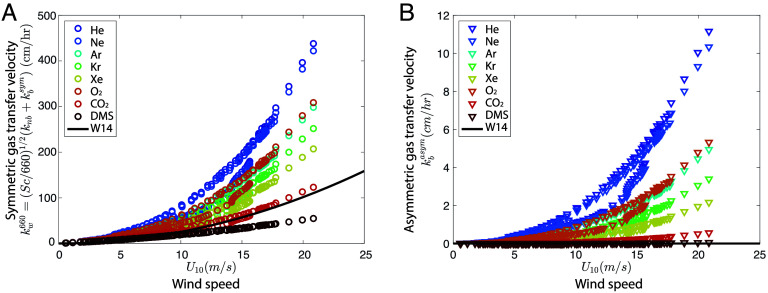
Wind–wave–bubble formulation of the gas transfer velocity (symmetric and asymmetric contributions) as a function of wind speed for a wide range of gases. (*A* and *B*) Transfer velocity from the derived wind–wave–bubble formulation (Eqs. **9** and **10**), shown as a function of wind speed. The wind and wave conditions are from the HiWinGS campaign with wind speed up to 25 m s^−1^ and significant wave height up to 4 m (29, 32). (*A*) Symmetric contribution (combining the nonbreaking and large bubbles), normalized by the Sc number, $k_{w}^{660}={\left(Sc/660\right)}^{1/2}\left(k_{\mathrm{nb}}+k_{b}^{\mathrm{sym}}\right)$. The symmetric contribution is identical for all gases in the classic wind-only formulation [Eq. **1**, W14 (17)], since it does not have an explicit solubility dependency (and the diffusivity scaling is accounted in the Sc normalization). (*B*) Asymmetric contribution $k_{b}^{\mathrm{asym}}$. The asymmetric contribution is zero in the wind-only formulation since it does not account for small bubbles. In both cases, the wind–wave–bubble-dependent formulation includes variability due to waves, with the same wind speed leading to different values in the gas transfer velocity for different wave fields.

The resulting symmetric and asymmetric wind–wave–bubble gas transfer velocity (Eqs. **9** and **10**) is illustrated in Fig. 3 *A* and *B* for a winter storm in the North Atlantic, for a wide range of gases (noble gases, CO_2_, O_2_, DMS). Fig. 3*A* shows the symmetric contribution of the gas transfer velocity $k_{w}^{660}={\left(Sc/660\right)}^{1/2}\left(k_{\mathrm{nb}}+k_{b}^{\mathrm{sym}}\right)$ (summing the surface and symmetric bubbles), comparing the wind–wave–bubble solubility-dependent formulation (Eq. **9**) to the classically used wind-only formulation of ref. 17 (black line W14). For each gas, waves introduce variability with the same wind speed leading to multiple values of the gas transfer velocity (Fig. 3, and discussed in refs. 30–32). For CO_2_, the values are relatively similar to the wind-only formulation (30–32). However, for gases such as O_2_, with a much lower solubility, the wind-only transfer velocity from ref. 17 is significantly lower than the wind–wave–bubble formulation, due to the solubility dependence in the large bubble symmetric gas transfer velocity. Fig. 3*B* shows the asymmetric gas transfer velocity for the same gases, which is zero in the wind-only formulation (solid line), with the O_2_ asymmetric contribution roughly an order of magnitude larger than the CO_2_ one, due to the strong dependence in solubility. As noted above, Eqs. **9** and **10** are valid for solubility α≲2, with the bubble contribution dropping dramatically for highly soluble gases (such as DMS), and becoming negligible (Fig. 2).

## Implications for the Ocean Oxygen Cycle

To demonstrate the importance of the asymmetric bubble flux for low solubility gases, we compare air–sea oxygen fluxes from the validated wind–wave–bubble formulation that incorporates sea state effects and injection of asymmetric bubbles with the classic wind-only formulation (17). The wind-only formulation ignores these effects but is used in almost all Earth System Models, including those analyzed for future ocean oxygen changes (e.g., refs. 7, 28, 54, and 55. We use a global ocean circulation model coupled with biogeochemistry (MOM6-COBALTv2; see *Materials and Methods*, refs. 8, 9, and 56 and compare two simulations: one in which we implement the three components of the wind–wave–bubble gas exchange for both CO_2_ and O_2_ fluxes (all formulae and coefficients are summarized in Table 1 and *SI Appendix*, Tables S1 and S2) and one with the classic wind-only formulation from ref. 17.

**Fig. 4.**
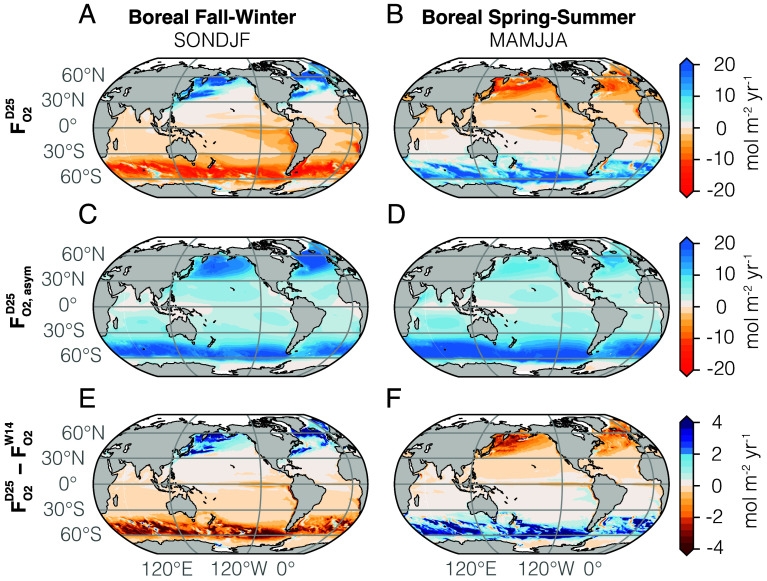
Boreal fall-winter (September–February) and spring-summer (March–August) averages (2006–2020, mol m^−2^ yr^−1^) of the air–sea O_2_ flux ($F_{O_{2}}$) using the wind–wave–bubble formulation in the MOM6-COBALTv2 model: (*A, B*) total $F_{O_{2}}$, (*C, D*) asymmetric term $F_{O_{2}}^{\mathrm{asym}}$, and (*E, F*) difference with the wind-only formulation (W14). Positive fluxes are into the ocean. Large differences up to 50% in local flux magnitude are visible at high latitudes between the two formulations.

Fig. 4 shows the northern hemisphere fall-winter (September to February) and spring-summer (March to August) averages of the oxygen flux obtained using the wind–wave–bubble gas transfer velocity including asymmetric bubbles, showing the total flux (*A* and *B*, summing all three components $F=F_{\mathrm{nb}}+F_{b}^{\mathrm{sym}}+F_{b}^{\mathrm{asym}}$), the asymmetric bubble flux (*C* and *D*, $F_{b}^{\mathrm{asym}}$), and the difference with the simulation using the wind-only formulation (*E* and *F*, $F-F^{W14}$). We observe a pronounced seasonal pattern with strong uptake of oxygen at mid- and high-latitudes in fall-winter (∼5 to 25 mol m^−2^ yr^−1^) and mild outgassing in the spring-summer hemisphere (∼−5 to −15 mol m^−2^ yr^−1^), while weaker fluxes are simulated in the tropics in both seasons (−5 to 5 mol m^−2^ yr^−1^, Fig. 4 and *SI Appendix*, Fig. S1). The wind-only formulation shows similar patterns, but the fall-winter flux is weaker, with local differences up to 50% at mid- and high-latitudes (i.e., enhanced uptake of up to 5 to 10 mol m^−2^ yr^−1^ in the wind–wave–bubble formulation in Fig. 4*C* and *SI Appendix*, Fig. S1), amounting to an additional fall-winter oxygen ocean uptake of about 79 Tmol yr^−1^ in the southern hemisphere and 32 Tmol yr^−1^ in the northern hemisphere in the wind–wave–bubble formulation (*SI Appendix*, Fig. S2). This stronger fall-winter uptake is partially offset by a stronger spring-summer outgassing of about 72 Tmol yr^−1^ in the southern hemisphere and 27 Tmol yr^−1^ in the northern hemisphere (Fig. 4 and *SI Appendix*, Fig. S2). This flux difference between formulations is directly caused by the bubble asymmetric term, which enhances the fall-winter oxygen uptake at mid- and high-latitudes (Fig. 4). This amplification of the air–sea flux seasonality (stronger uptake in fall-winter and stronger outgassing in spring-summer) partially compensates in the global annual average (*SI Appendix*, Fig. S1), but it is likely to influence oxygen patterns in the ocean interior. For instance, the stronger fall-winter oxygen uptake is expected to affect interior ocean properties (deeper mixed layers) than the stronger spring-summer outgassing (shallower mixed layers).

The globally integrated annual flux can be computed for wind-only and wind–wave–bubble formulations. The globally integrated ocean oxygen loss in the wind–wave–bubble formulation accounting for asymmetric bubbles is weaker than in the wind-only purely symmetric formulation, with a significant magnitude difference of about 12 Tmol yr^−1^ (−48 Tmol yr^−1^ in wind-only vs. −36 Tmol yr^−1^ in wind–wave–bubble, over 2006–2020). These results on global air–sea oxygen flux can be compared to observation-based oxygen inventory change estimate from ref. 27, which uses the World Ocean Database (57) to provide dissolved oxygen changes and associated uncertainties for the global ocean using an optimal interpolation method applied to quality-controlled bottle O_2_ data. The oxygen loss in the wind-only case (−48 Tmol yr^−1^) is larger than the range of the observation-based estimate (−28 to −37 Tmol yr^−1^ over 1965–2020, 95% confidence level), while the wind–wave–bubble case (−36 Tmol yr^−1^) lies within the range of this observation-based estimate, suggesting that the inclusion of the bubble asymmetric flux is key to evaluate global ocean oxygen loss and might reconcile some of the discrepancies between observed and modeled oxygen trends (58, 59).

**Fig. 5.**
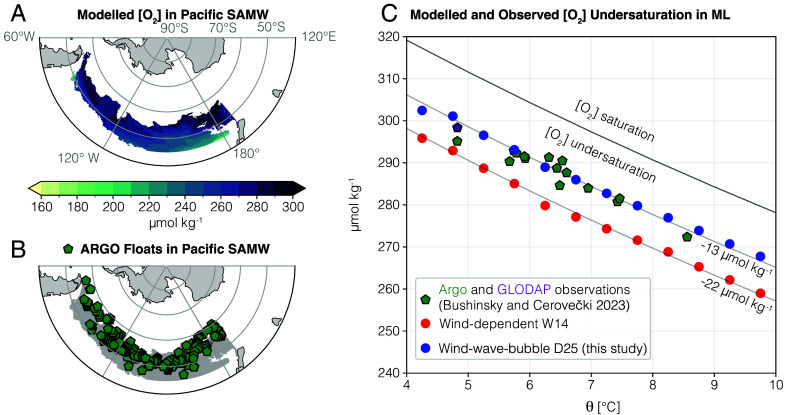
Observed and modeled oxygen concentrations in the Pacific Subantarctic Mode Waters (SAMW) during wintertime formation (August–September). (*A*) Model annual mean oxygen concentrations in the Pacific SAMW detected following the observational criteria used for the Argo floats (41), i.e., using mixed layer depth deeper than 200 m and potential density $26.8\leq \sigma_{\theta }<27.05$ kg m^−3^(where colors are present). (*B*) Argo float locations (green pentagons) overlapping with the SAMW detected in the model. (*C*) Annual mean SAMW wintertime mixed layer oxygen concentration as a function of potential temperature for in situ observations from GLODAP (purple pentagon) and Argo (green pentagons) (41), and medians in bins of temperature for the ocean model simulations with the two gas exchange formulations: wind-only (red) and wind–wave–bubble (blue). Oxygen saturation and undersaturation by 13 and 22 μmol kg^−1^ (gray lines). The wind–wave–bubble formulation leads to increased uptake and improved representation of the oxygen concentration.

We further compare the oxygen concentration in the Southern Ocean in the ocean model to recent in situ observations from Argo biogeochemical profiling floats (60, 61) and shipboard measurements from the Global Ocean Data Analysis Project [GLODAPv2.2020, (62, 63)]. We consider Pacific Subantarctic Mode Waters (SAMW) during their formation period in winter (August–September) when they outcrop in the Southern Ocean between 50^°^S and 60^°^S and are in contact with the atmosphere under the strong influence of air–sea exchange (41). Pacific SAMW are detected in the ocean model and the observations following ref. 41, using criteria of potential density and mixed layer depths (>200 m) in the Pacific sector of the Southern Ocean (see Fig. 5*A* and *Materials and Methods*). Fig. 5*B* shows the oxygen concentration from the Argo and shipboard in situ observations (green pentagons) compared to the ocean model using the wind-only (red) and the wind–wave–bubble (blue) formulations as a function of ocean temperature. In their analysis of in situ observations, ref. 41 noted that oxygen concentrations in newly formed SAMW showed a consistent degree of undersaturation despite significant interannual variability (oxygen range of about 270 to 300 μmol O_2_ kg^−1^ for temperatures of 5 to 8.5 ^°^C, falling below the saturation curve shown a by solid line in Fig. 5*B*). This systematic undersaturation by about 13 μmol kg^−1^ arises from a relatively constrained balance between air–sea oxygen fluxes (controlled by surface heat loss increasing solubility) and the entrainment of deep, oxygen-deficient waters from below (controlled by surface heat loss and convective mixing).

The simulation with the wind–wave–bubble formulation captures remarkably well the magnitude of the observed undersaturation (blue circles align with green pentagons, Fig. 5*B*). In contrast, the simulation with the wind-only formulation systematically underestimates the oxygen concentration, yielding levels of undersaturation of around 22 μmol kg^−1^, much higher than in observations and the wind–wave–bubble simulation (Fig. 5*B*). The new wind–wave–bubble formulation injects significantly more oxygen into SAMW during their wintertime formation than the wind-only formulation, leading to an improved agreement with the in situ data. After their formation, SAMW are transported by ocean circulation and ventilate the ocean interior (41). This result indicates that the oxygen concentration and distribution in models using a nonbubble resolving gas flux formulation are likely to be biased, with implications when they are used for projections under climate change.

## Conclusion

We have provided a theoretical framework to describe gas exchange for a wide range of gas diffusivity and solubility, incorporating turbulence enhanced diffusive gas exchange at the surface together with large bubbles spending a finite amount of time in the water and getting squeezed by hydrostatic pressure as well as small bubbles fully dissolving in the water column. Small and large squeezed bubbles induce an asymmetric gas exchange, with gas always going into the water column, and are usually not considered in large-scale ocean circulation modeling. For bubbles, an explicit dependence on wind and waves is presented, accounting for wave breaking statistics and constraining the total amount of air being entrained, combined with a bubble-mediated gas exchange theory. The theory considers the injected depth, the bubble size distribution, the bubble rise velocity and individual exchange velocity and leads to a different dependency in solubility and diffusivity for the large and small bubbles, which is in agreement with laboratory experiments reporting noble gas supersaturation at high wind speed, in a bubble saturated environment.

From the theory, we derive a simpler wind–wave–bubble formulation for both the symmetric and asymmetric bubble contribution that depends on wind speed, significant wave height, gas solubility, and diffusivity. The new formulation is easy to use as the required wave products are available alongside the wind products (31, 32). The wind–wave–bubble formulation retains sea state information but can also be further simplified to only depend on wind speed (51), by expressing the air entrainment (analog to the whitecap coverage) as a function of wind speed only (instead of wind and significant wave height), and keeping the specific dependency in solubility and diffusivity of the symmetric and asymmetric bubble gas transfer. The formulae are provided in *Materials and Methods* and *SI Appendix*, Table S1, and we provide scripts to compute the wind–wave–bubble formulation. This even more simplified approach depends on wind-only (and does not incorporate the sea state induced variability), but unlike the widely used wind-only formulations, it represents the mean bubble asymmetric contribution critical to low solubility gases such as oxygen.

The wind–wave–bubble gas exchange formulation proposed here was implemented in a global ocean-biogeochemical model. We discuss the oxygen fluxes and the difference with the classic wind-only formulation that does not account for the small asymmetric bubbles, highlighting increased uptake during fall-winter and reduced outgassing during spring-summer at mid- and high-latitudes. We compare the resulting annual oxygen flux against oxygen loss inventory as well as the oxygen concentration in wintertime in the Southern Ocean from recent in situ measurements. In both cases, the inclusion of the bubble asymmetric flux leads to an improved agreement between the ocean model and in situ observations which suggests that our formulation provides a path to improve the representation of low solubility gases such as oxygen in large-scale ocean and climate models, with broad implications for biogeochemical cycles and marine ecological modeling.

## Materials and Methods

### Wind-Only Gas Transfer Velocity.

The classic wind-only formulation (W14, ref. 17) used together with eq. 1 in most ocean and climate models and observation-based flux products, is given by

[11] $$\begin{array}{c} k_{w}^{W14}=A_{W14}U_{10}^{2}{\left(\mathrm{Sc}/660\right)}^{-1/2}, \end{array}$$

where $A_{W14}=0.251$ (cm hr^−1^) (m s^−1^)^−2^) is an empirical coefficient adjusted to match radiocarbon budget (14, 17, 64), passive tracer fluxes (15, 16) or eddy covariance CO_2_ fluxes (18).

### Bubble Model Equations.

The bubble model requires knowledge of the bubble size distribution, rise velocity, individual bubble exchange coefficient and injection depth. The efficiency coefficient is written in terms of the depth of bubble injection $z_{0}$, and an equilibration depth $H_{\mathrm{eq}}\left(R_{b}\right)$, which can be interpreted as the depth at which the bubble will exchange all of its gas content,

[12] $$\begin{array}{c} E\left(R_{b}\right)=\frac{z_{0}}{z_{0}+H_{\mathrm{eq}}\left(R_{b}\right)},\text{and}\;H_{\mathrm{eq}}\left(R_{b}\right)=\frac{4\pi }{3\alpha }\frac{R_{b}w_{b}\left(R_{b}\right)}{\kappa_{b}\left(R_{b}\right)}, \end{array}$$

where $w_{b}\left(R_{b}\right)$ and $\kappa_{b}\left(R_{b}\right)$ are the individual bubble rise velocity and exchange rate; and $z_{0}$ the bubble injection depth. The injection depth is proportional to the breaking height and is taken as $z_{0}\approx 0.5$ m in the laboratory configuration at high wind speed (sensitivity tests are shown in *SI Appendix*, Figs. S7 and S8); and the bubble cut-off size is $R_{\mathrm{inj}}=150\;\mu$m. We consider the rise velocity and individual transfer rates derived for individual bubbles (4, 5, 30) see *SI Appendix*.

The hydrostatic pressure term acting on large bubbles, used in the asymmetric gas transfer velocity is written as

[13] $$\begin{array}{c} \frac{\Delta P}{P_{0}}\frac{V_{\mathrm{exch}}}{\alpha }=\frac{1}{\alpha }\int dR_{b}\left(4\pi /3\right)R_{b}^{3}Q\left(R_{b}\right)F\left(R_{b}\right), \end{array}$$

[14] $$\begin{array}{c} F\left(R_{b}\right)=\frac{H_{\mathrm{eq}}}{H_{0}}\frac{z_{0}^{2}}{{\left(z_{0}+H_{\mathrm{eq}}\left(R_{b}\right)\right)}^{2}}, \end{array}$$

where $P_{0}$ is the total atmospheric pressure and $H_{0}=P_{0}/\left(\rho g\right)$.

Note that the obtained gas transfer velocity shown in Fig. 3 *A* and *C* is of comparable value to the high wind speed gas transfer velocity from ref. 65 in forced evasion experiments.

The bubble induced supersaturation can also be estimated (5). The supersaturation is defined as the excess concentration in the water and has both exchanged and injected components, $\Delta =\Delta^{\mathrm{sym}}+\Delta^{\mathrm{asym}}$, with $\Delta^{\mathrm{asym}}=\frac{V_{\mathrm{inj}}/\alpha }{k_{\mathrm{nb}}+k_{b}^{\mathrm{sym}}}$, and $\Delta^{\mathrm{sym}}=\frac{\Delta P}{P_{0}}\frac{k_{b}^{\mathrm{sym}}}{k_{\mathrm{nb}}+k_{b}^{\mathrm{sym}}}$, so that the total bubble induced supersaturation reads

[15] $$\begin{array}{c} \Delta =\frac{k_{b}^{\mathrm{asym}}}{k_{\mathrm{nb}}+k_{b}^{\mathrm{sym}}}. \end{array}$$

### Wave and Ocean Modeling.

We use the ocean circulation model from the Geophysical Fluid Dynamics Laboratory (GFDL) (8) global ocean model (Modular Ocean Model, MOM6) (56) coupled with sea ice and biogeochemistry (COBALTv2) (9). The model has a nominal 0.5° grid and includes 75 hybrid vertical layers, with grid spacing of about 2 m near the surface and a modified potential density coordinate at depth. The ocean model was spun up [using the wind-only formulation from Wanninkhof et al. (17)] for 135 y, after which the modeled interactive chlorophyll self-shading feedback was shut off for 30 y and replaced by the MOM6 physics-only chlorophyll self-shading feedback to simplify the comparison of different simulations (the interactive feedback can impact ocean mesoscale dynamics and change the position of eddies), and atmospheric pCO_2_ was ramped up from preindustrial conditions (66). Hindcast simulations for the wind–wave–bubble formulation including asymmetric bubbles are activated in 1959 and ran until 2020, and uses the NCAR momentum flux formulation. We analyze the 2006–2020 period corresponding to the period with Argo observational constraints (40).

We use sea state and significant wave heights from the global spectral wave model WAVEWATCH III [WW3, (67)] described previously in ref. 32. WW3 simulates the growth and propagation of wave energy of the wave spectrum, for a given wind forcing. The global WW3 and the hindcast formulations are forced by the Japanese Meteorological Society Reanalysis product (JRA55-do v1.5), which provides 10 m wind vectors ($U_{10}$) at approximately half-degree resolution every three hours (68, 69). Air entrainment $V_{A}$ required for the full gas transfer formulation was evaluated using breaking statistics (6, 30, 49), itself evaluated through the wave spectrum model from ref. 50 following previous work (32, 51). The wave model produces output of the wave spectrum, from which the significant wave height $H_{s}$ required for the simpler wind–wave–bubble formulation can be computed. Here, we used the $H_{s}$ computed globally and available for public use as published by Zhou et al. (32).

### Wind-Only Formula Including Bubble Asymmetric Flux.

The wind–wave–bubble formulation can be further simplified to only consider wind speed (51). The air entrainment (analog to the whitecap coverage) follows approximately $V_{A}\propto u_{*}^{5/3}{\sqrt{gH_{s}}}^{4/3}\propto {\left(U_{10}-2.5\right)}^{2.5}$, so that $k_{b}^{w,sym}=A_{w,b}{\left(U_{10}-2.5\right)}^{2.5}{\left(Sc/660\right)}^{-1/2}\alpha^{-0.35}$; and $k_{b}^{w,asym}=A_{w,asym}\alpha^{-0.65}{\left(U_{10}-2.5\right)}^{2.5}$ (above 2.5 m s^−1^, with 0 below, and up to typically 25 m s^−1^). The associated coefficients are $A_{w,b}=0.012A_{b}$ and $A_{w,asym}=0.012A_{\mathrm{asym}}$. We note that the specific values of the coefficients depend on the momentum flux (drag coefficient) formulation (NCAR and COARE 3.5 are shown in *SI Appendix*, Fig. S5). The summary of formulae and coefficients are in *SI Appendix*, Tables S1 and S2 for two commonly used drag formulations and the wind-only formulation is also shown in *SI Appendix*, Fig. S6, retaining all three flux components, and their dependency on diffusivity and solubility.

## Supplementary Material

Appendix 01 (PDF)

## Data Availability

The WAVEWATCH III model is available at: https://github.com/NOAA-EMC/WW3; and with the (50) formulation for breaking distribution and volume of air entrained is available at: https://github.com/Leonel-Romero/WW3-Lambda. Scripts to compute the wind–wave–bubble formulation are available at https://github.com/paridhirustogi10/D25_windwavebubble and https://zenodo.org/records/14961862. Wind-wave data are provided in refs. 31 and 32.
