# Supplementary material for: A universal wind–wave–bubble formulation for air–sea gas exchange and its impact on oxygen fluxes
Source: Proc Natl Acad Sci U S A. 2025 Sep 16;122(38):e2419319122. doi: 10.1073/pnas.2419319122 (PMC12478149; doi:10.1073/pnas.2419319122)
Supplement: Supplementary file 1 — Appendix 01 (PDF) [file pnas.2419319122.sapp.pdf]

PNAS
www.pnas.org

Supplementary Information for

A universal wind-wave-bubble formulation for air-sea gas exchange and its impact on oxygen fluxes.

Luc Deike, Xiaohui Zhou, Paridhi Rustogi, Rachel H. R. Stanley, Brandon G. Reichl, Seth M. Bushinsky and Laure Resplandy

Luc Deike.
E-mail: ldeike@princeton.edu

This PDF file includes:

Figs. S1 to S8
Tables S1 to S2
References for SI reference citations

1. Summary of model coefficients, for various wind-wave drag coefficients, and only keeping the wind dependence

Wind-wave-bubble formulation using COARE 3.5 for $u_* - U_{10}$ relationship

	Non-breaking	Symmetric bubbles	Asymmetric bubbles
Flux (mol m^{-2} s $^{-1}$)	$F_{nb} = k_{nb} S(P_a - P_w)$	$F_b^{sym} = k_b^{sym} S(P_a - P_w)$	$F_b^{asym} = k_b^{asym} S P_a$
Transfer velocity (m s^{-1})	$k_{nb} = A_{nb} \frac{u_*}{(Sc/660)^{1/2}}$	$k_b^{sym} = A_b \frac{u_*^{5/3} \sqrt{gH_s}^{4/3}}{\alpha^{0.35} (Sc/660)^{1/2}}$	$k_b^{asym} = A_{asym} \frac{u_*^{5/3} \sqrt{gH_s}^{4/3}}{\alpha^{0.65}}$
Coefficients	$A_{nb} = 1.33 \cdot 10^{-4}$	$A_b = 1.2 \cdot 10^{-5}$ (m^{-2}s^{-2})	$A_{asym} = 7 \cdot 10^{-8}$ (m^{-2}s^{-2})
Uncertainty	$\pm 0.1 \cdot 10^{-4}$	$\pm 0.1 \cdot 10^{-5}$ (m^{-2}s^{-2})	$\pm 4 \cdot 10^{-8}$ (m^{-2}s^{-2})
Theory constraints	Eddy renewal	Air entrained and Bubble model	Air entrained and Bubble model
Data constraints	Field CO_2 and DMS fluxes at low wind speed	Field CO_2 fluxes at moderate to high wind speed	Lab and field supersaturation of noble gases

Wind-wave-bubble formulation using NCAR $u_* - U_{10}$ relationship

	Non-breaking	Symmetric bubbles	Asymmetric bubbles
Flux (mol m^{-2} s $^{-1}$)	$F_{nb} = k_{nb} S(P_a - P_w)$	$F_b^{sym} = k_b^{sym} S(P_a - P_w)$	$F_b^{asym} = k_b^{asym} S P_a$
Transfer velocity (m s^{-1})	$k_{nb} = A_{nb} \frac{u_*}{(Sc/660)^{1/2}}$	$k_b^{sym} = A_b \frac{u_*^{5/3} \sqrt{gH_s}^{4/3}}{\alpha^{0.35} (Sc/660)^{1/2}}$	$k_b^{asym} = A_{asym} \frac{u_*^{5/3} \sqrt{gH_s}^{4/3}}{\alpha^{0.65}}$
Coefficients	$A_{nb} = 1.38 \cdot 10^{-4}$	$A_b = 1.6 \cdot 10^{-5}$ (m^{-2}s^{-2})	$A_{asym} = 7 \cdot 10^{-8}$ (m^{-2}s^{-2})
Uncertainty	$\pm 0.1 \cdot 10^{-4}$	$\pm 0.1 \cdot 10^{-5}$ (m^{-2}s^{-2})	$\pm 4 \cdot 10^{-8}$ (m^{-2}s^{-2})

Wind-only formula including bubble asymmetric flux

	Non-breaking	Symmetric bubbles	Asymmetric bubbles
Flux (mol m^{-2} s $^{-1}$)	$F_{nb} = k_{nb} S(P_a - P_w)$	$F_b^{sym} = k_b^{sym} S(P_a - P_w)$	$F_b^{asym} = k_b^{asym} S P_a$
Transfer velocity (m s^{-1})	$k_{nb} = A_{nb} \frac{u_*}{(Sc/660)^{1/2}}$	$k_b^{sym} = \tilde{A}_b \frac{(U_{10} - 2.5)^{2.5}}{\alpha^{0.35} (Sc/660)^{1/2}}$	$k_b^{asym} = \tilde{A}_{asym} \frac{(U_{10} - 2.5)^{2.5}}{\alpha^{0.65}}$
Coefficients	$A_{nb} = 1.33 \cdot 10^{-4}$	$\tilde{A}_b = 1.42 \cdot 10^{-7}$ (m$^{-5/2}$s$^{-5/2}$)	$\tilde{A}_{asym} = 1.18 \cdot 10^{-9}$ (m$^{-5/2}$s$^{-5/2}$)

Table S1. Wind-wave-bubble formulation for gas exchange: non-breaking, symmetric and asymmetric bubbles contributions. Top: shows values obtained using the COARE 3.5 momentum flux/drag coefficient, and recall the various data and theoretical constraints to link u_* and U_{10}, (same information in Table 1 from the main text). Middle: shows the same formulation but converting the coefficients when using NCAR momentum flux formulation to link u_* and U_{10}. (leading to the same gas transfer velocity results as the set of coefficients obtained when using COARE 3.5). These coefficients are used in MOM6-COBALTv2 model since it uses the NCAR parameterization for the momentum flux/drag coefficient. Bottom: shows the same formulation but now converting the wind-wave-bubble formulation into a wind-wave-bubble based on wind speed only formulation (retaining the separation into three flux components). Coefficients are given to yield transfer velocity in m s^{-1} (which are then converted to cm hr^{-1} in the plots following the usual convention in the literature).

Input variables

Short name (units)	Long Name	Ocean model analysis	SUSTAIN laboratory analysis
U_{10} (m s^{-1})	Wind speed at 10 m	Reanalysis (JRA55-do v1.5) (1, 2)	Measured
H_s (m)	Significant wave height	Wave model WW3 (3, 4)	Measured
T (K)	Sea Surface Temperature	ocean model	Measured
Salinity	Sea Surface Salinity	ocean model	Measured
P_a	Atmospheric pressure / Gas partial pressure	Reanalysis (JRA55-do v1.5) (1, 2)	Measured
P_w	Gas partial pressure water	ocean model	Measured

Formulation constants

A_{nb}	Non-breaking prefactor	Data and theory: Deike and Melville 2018, Zhou et al 2023 and this paper
A_b	Bubble symmetric prefactor	Data and theory: Deike and Melville 2018, Zhou et al 2023 and this paper
A_{asym}	Bubble asymmetric prefactor	Data and theory: This paper

Calculated variables

u_* (m s^{-1})	Wind friction velocity	Momentum flux (drag coefficient) formulation NCAR (5) or COARE (6)
S (mol s^2 kg^{-1} m^{-2})	Solubility	Tabulated function of temperature (7)
α	Ostwald Solubility (dimensionless)	$\alpha = RTS$
R (kg m^2 s^{-2} mol^{-1}K^{-1})	Ideal gas constant	R=8.31 (kg m^2 s^{-2} mol^{-1}K^{-1})
D (m^2 s^{-1})	Gas Diffusivity in water	Tabulated function of temperature and salinity (8)
ν (m^2 s^{-1})	Water kinematic viscosity	Tabulated function of temperature (9)
Sc	Schmidt number (dimensionless)	$Sc = \nu / D$

Table S2. Variables used in gas flux formulation, and how we estimated them in the ocean model and in the laboratory experiments. Note that other choices of input reanalysis products for the wind could be used (such as ERA5) and wave model.

Table S1 summarizes the wind-wave-bubble flux formulation coefficients, theoretical and empirical constraints, for two different momentum flux/drag coefficients formulations commonly used (COARE 3.5 and NCAR). The corresponding formulation for

the further simplified formula expressed only in terms of the wind (and not the waves) retaining the bubble flux components is also provided. Uncertainties are estimated based on the comparison to field observations of CO2 and DMS fluxes for A_{nb} and A_b; and sensitivity to the model parameters for A_{asym} (since no direct measurements of low solubility gases fluxes in the field are available).

Table S2 summarizes the variables used in the gas transfer formulations (provided in table S1), with details over the input variables and how they are computed in the ocean model and laboratory, the formulation constants and the calculated variables.

2. Oxygen fluxes maps (2006-2020)

Oxygen fluxes are computed from the coupled MOM6-COBALTv2 runs, using the new wind-wave-bubble formulation (D25), the classic wind-only formulation (W14), and their difference (D25-W14) over the 2006-2020 period.

Figure S1 shows maps of the oxygen flux with the annual mean (first row a-c), the boreal fall and winter average (second row d,e,f), and the boreal spring and summer (third row g-i). The first column shows the fluxes with the wind-wave-bubble formulation (D25), the second column shows the fluxes with the wind-only formulation (W14), and the third column shows the difference.

Figure S2 shows the fluxes integrated over all longitude and as a function of the latitude with the annual mean (a), the boreal fall and winter average (b), and the boreal spring and summer (c); together with a table summarizing the integrated fluxes over the Northern and Southern Hemisphere for each season and in total.

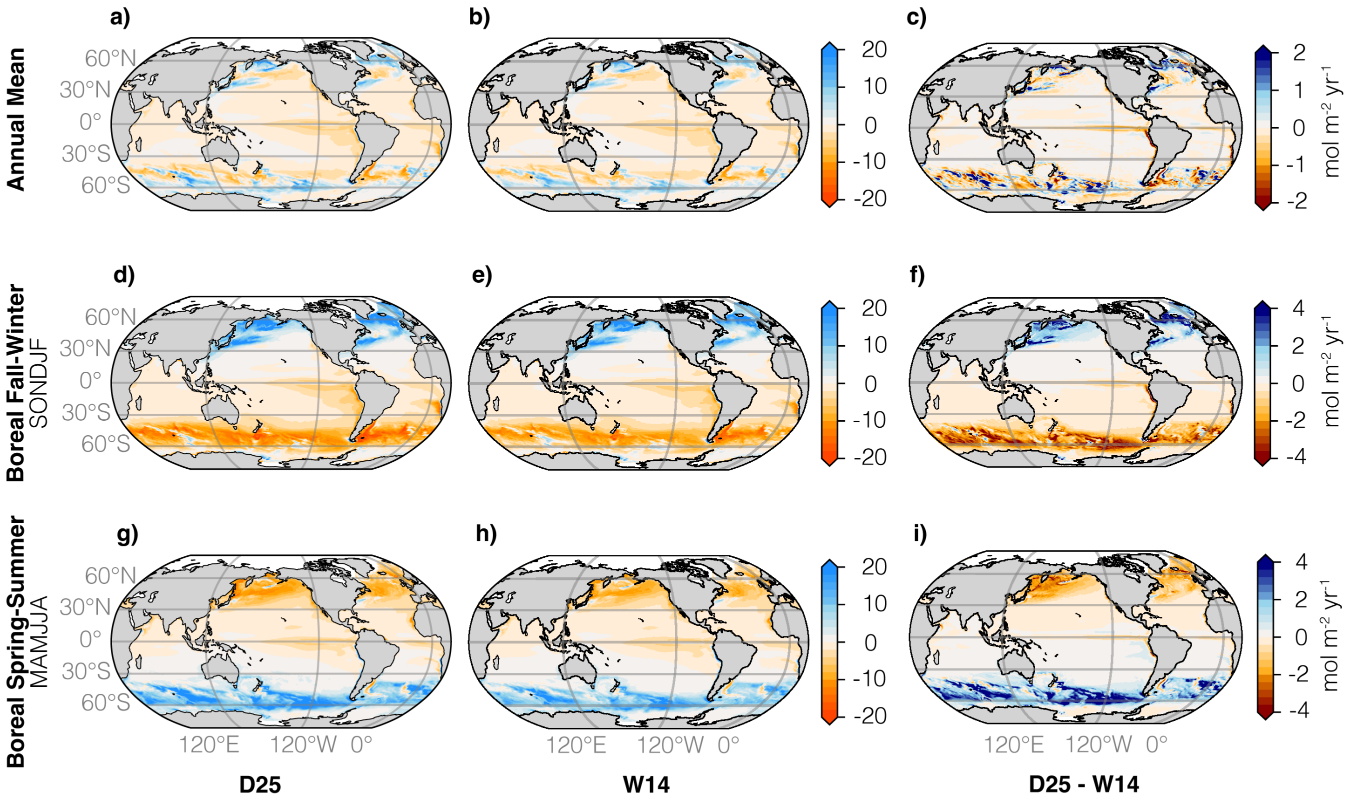

Fig. S1. Seasonal and annual average of the oxygen fluxes from the wind-wave-bubble formulation (D25, left column); wind-only formulation (W14, middle column); and the difference between the two (right column). Top row shows the annual average. Middle row is the boreal fall and winter average (September-February), and bottom row is the boreal spring and summer (March-August). Note that we show the total flux (so for the wind-wave-bubble formulation $F_{nb} + F_b^{sym} + F_b^{asym}$.

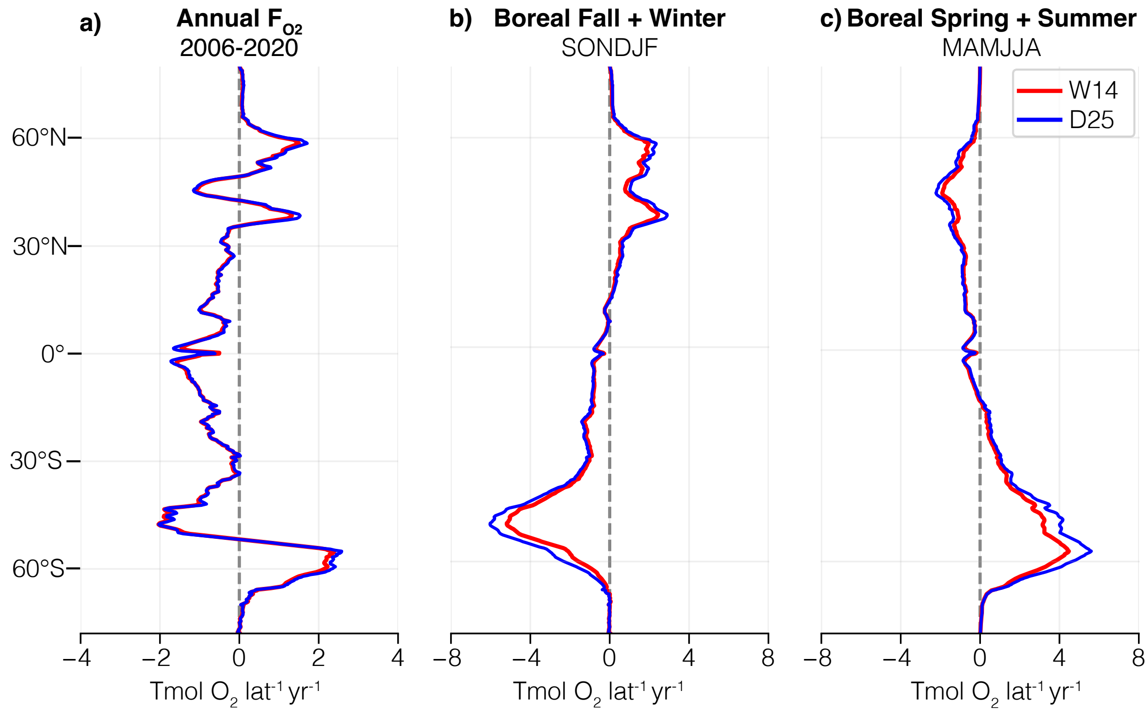

	Tmol O_2 yr^{-1}	SONDJF	MAMJJA	Total		SONDJF	MAMJJA	Total
D25	Northern Hemisphere	183	-187	**-4**	D25 - W14	32	-27	**5**
	Southern Hemisphere	-418	386	**-32**		-72	79	**7**
	Total	**-235**	**199**	**-36**		**-40**	**52**	**+12**

Fig. S2. Oxygen flux integrated over longitudes, and over the 2006-2020 period. Blue lines indicate our new wind-wave-bubble formulation (D25) accounting for the asymmetric bubble component while red is the wind-only formulation (W14). a) annual flux, b) boreal fall and winter (September to February) and c) boreal spring and summer (March to August). The table below the plot indicates the integrated flux in the Northern and Southern Hemispheres for both seasons in Tmol yr^{-1} of oxygen.

3. Comparison of the bubble induced predicted supersaturation to data from Stanley et al 2009

The order of magnitude predicted bubble induced supersaturation can be compared to noble gas measurements from the BATS data set (10). Figure S3 shows the supersaturation in He compared to the predicted supersaturation as a function of wind speed. Wind speed is obtained from the JRA55-do v1.5 product, significant wave height from WAVEWATCH III run and SST to compute solubility and diffusivity from the ocean model. The same choice of coefficient of $A_{asym} \approx 7 \cdot 10^{-8}$ is used and provides a reasonable agreement between the reported supersaturation and the bubble supersaturation. The uncertainty in the binned data is relatively large so that only an order of magnitude comparison is made. The He supersaturations from the BATS data set were scaled down by 2% because the at-sea extraction method used for extracting gases from those samples led to erroneously high supersaturations. The correction factor was determined by quantitatively comparing the average of all the Ne data below 2000 m in the three year BATS time series to the average of recent deep Ne Atlantic data (11).

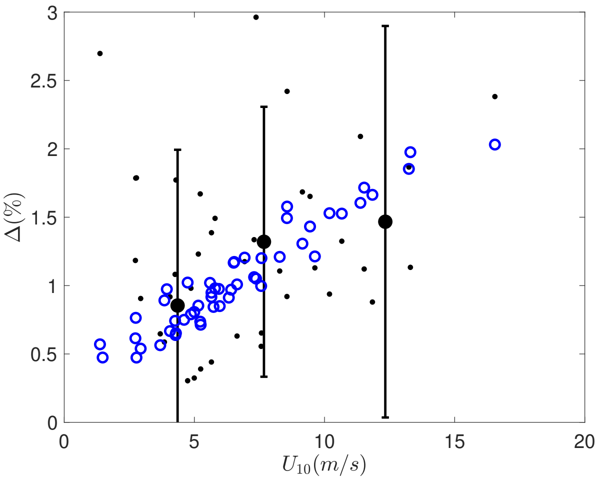

Fig. S3. Comparison between He supersaturation from the Bermuda described in Stanley et al., (2009) (12) against the wind-wave-bubble formulation derived from theory and experiments proposed in this paper. Individual small Black dots are individual data while larger black full circles are data binned by wind-speed. Blue circles are obtained from the wind-wave bubble formulation.

4. Bubble-mediated gas transfer theory

All bubble mediated gas transfer models require information on the injection depth (z_0), rise velocity ($w_b(R_b)$), individual bubble exchange rate ($\kappa_b(R_b)$), as well as the bubble size distribution $Q(R_b)$ (which will depend on wind speed and wave height). We recall here all the formulae already discussed in (4, 13) and used in the main text and figures.

A. Bubble injection depth, rise velocity and individual gas transfer. The injection depth can be estimated as the breaking height, $z_0 \propto h$, supported by laboratory experiments and simulations (14–18), and some field measurements (19, 20). The rise velocity and individual transfer rates derived for bubbles moving in quiescent flow have been used, for clean and contaminated (dirty) interfaces (21, 22). As discussed in Keeling (22), the choice of entrained depth z_0 has an influence on the final scaling of the symmetric and asymmetric gas transfer velocity with the solubility α (and diffusivity D). In the laboratory experiments at high wind speed (figure 2 in the main paper), we consider $z_0 = 0.5$m guided by experimental observations of air entrainment at high wind speed. Sensitivity tests are presented in figure S7 as illustration while $z_0 = 0.5$m is the most physical choice (given the height of the waves in the high wind speed conditions). In open ocean conditions, we typically use $z_0 \propto H_s/2$ (13).

In the original (22) model, the rise velocity in quiescent water, following (23) is used, which consider the rise velocity in dirty water, as contaminants are present in ocean water and reduce the rise velocity of the bubbles

$$w_b(r) = \frac{2r^2 g}{9\nu_w}\left[(\upsilon^2 + 2\upsilon)^{1/2} - \upsilon\right], \text{ with } \upsilon = 10.82/\zeta, \text{ and } \zeta = \frac{gr^3}{\nu_w^2}. \quad [1]$$

Similarly, the individual transfer velocity of the bubbles used corresponds to a bubble rising at velocity w_b in a quiescent flow, considering either a clean (22) or dirty interface (21), $\kappa_b(r) = 8\sqrt{\frac{\pi D w_b(r)}{2r}}$. We note that rise velocity and individual transfer rate could be modified in turbulence (24).

B. Bubble size distribution under a breaking wave. The bubble size distribution entrained by a breaking wave has received extensive scrutiny and the one considered here is similar to the one described in previous work (4, 13, 24). Based on laboratory experiments (15, 25, 26) and numerical simulations (16, 27–29), the bubble size distribution can be described as two power laws, above and below the Hinze scale R_H (30). The Hinze scale is the scale at which surface tension balances turbulence deformation and in typical upper ocean conditions, $R_H \approx 1$mm (15). The size distribution $Q(R_b)$ of bubbles entrained under a breaking wave, and defined as the number of bubbles per bin size, per unit volume, follows $Q(R_b) \propto R_b^{-10/3}$ for bubbles $R_b > R_H$, while $Q(R_b) \propto R_b^{-3/2}$ for $R_b < R_H$ (15, 27–29). The maximum bubble size considered is $R_{max} = 10$mm and the minimum bubble size is 10μm (as in (4, 13). Changing R_{min}, R_{max} within less than a factor 2 (which corresponds to variations suggested by laboratory data (24)) is negligible. Physical arguments have been proposed to describe both regimes, due to turbulent fragmentation (31), and capillary induced fragmentation (29) The total number of entrained bubbles is constrained by the breaker geometry (wave slope and wave speed) and a balance between buoyancy and turbulence forces (16, 27). Systematic field measurements of the bubble size distribution under breaking waves close to the water surface are yet to be realized due to significant technical challenges (32, 33).

C. Exchanged volume and injection volume. For completeness, we present here all equations used to compute the bubble transfer velocities (with the key equations already presented in the main paper and methods). The complete derivation can be found in Keeling 1993 (22).

The size distribution is integrated to provide the exchanged volume (made of bubbles above a cut-off size R_{inj}) and the injection volume (made of the smaller bubbles). The exchanged volume (given in the main text eq. 7), is calculated as:

$$V_{exch} = \int_{R_{inj}}^{R_{max}} dR_b (4\pi/3) R_b^3 Q(R_b) E(R_b), \qquad [2]$$

The injected volume is calculated as:

$$V_{inj} = \int_{R_{min}}^{R_{inj}} Q(R_b) 4/3\pi R_b^3 dR_b \qquad [3]$$

The size distribution $Q(R_b)$ is given in 4.B of the SM. We consider (in figure 2 in the paper) $R_{inj} = 150\mu$m and $R_{min} = 10\mu$m. The sensitivity between 50 and 250 microns to R_{inj} (the range estimated to balance rise velocity and turbulent fluctuations (24)) is illustrated later in fig. S8. The injected volume (eq. 3 in SI) is sensitive to the value of R_{inj} and will therefore modify the asymmetric gas transfer velocity (and as a consequence the supersaturation). The choice of $R_{inj} = 150\mu$m leads very good agreement between predicted and observed supersaturation and is coherent with the balance between turbulence fluctuations and rise velocity in the high wind speed conditions, as discussed in the main paper and methods. We recall the efficiency factor provided in the main paper (eq. 12 in the main paper):

$$E(R_b) = \frac{z_0}{z_0 + H_{eq}(R_b)}, \text{ and } H_{eq}(R_b) = \frac{4\pi}{3\alpha}\frac{R_b w_b(R_b)}{\kappa_b(R_b)}, \qquad [4]$$

where $w_b(R_b)$ and $\kappa_b(R_b)$ are the individual bubble rise velocity and exchange rate (given in 4.A); and z_0 the bubble injection depth. Sensitivity to z_0 is illustrated in fig. S7.

From the above equations, the injected volume is constant across gases (different solubility and diffusivity) while the exchanged volume will depend on the solubility and diffusivity through the integral over the efficiency term. Both terms depend on the wind speed (and wave height) through the entrained bubble size distribution $Q(R_b)$. Next, the overpressure term (used to compute the asymmetric contribution coming from large exchanged bubbles) is

$$\frac{\Delta P}{P_0} = \frac{\int_{R_{inj}}^{R_{max}} dR_b (4\pi/3) R_b^3 Q(R_b) F(R_b)}{\int_{R_{inj}}^{R_{max}} dR_b (4\pi/3) R_b^3 Q(R_b) E(R_b)}, \qquad [5]$$

with

$$F(R_b) = \frac{H_{eq}}{H_0}\frac{z_0^2}{(z_0 + H_{eq}(R_b))^2}, \qquad [6]$$

where P_0 is the total atmospheric pressure and $H_0 = P_0/(\rho g)$. The terms V_{exch} and $\Delta P/P_0$ are therefore functions of solubility and diffusivity, with a scaling that will depend on parameter choices (z_0, bubble size distribution, cut-off sizes).

From the above equations, the symmetric and asymmetric bubble gas transfer velocity are calculated (eq. 7 and 8 in the main text repeated here):

$$k_b^{sym} = \frac{V_{exch}}{\alpha} = \frac{1}{\alpha}\int_{R_{inj}}^{R_{max}} dR_b (4\pi/3) R_b^3 Q(R_b) E(R_b), \qquad [7]$$

$$k_b^{asym} = \frac{V_{inj}}{\alpha} + \frac{\Delta P}{P_0}\frac{V_{exch}}{\alpha}, \qquad [8]$$

where the asymmetric contribution coming from the exchanged bubbles (second term on the right hand side) can be written combining eq. 2 and 5 from the SI (and already given in methods of the main paper, eq. 13).

$$\frac{\Delta P}{P_0}\frac{V_{exch}}{\alpha} = \frac{1}{\alpha}\int_{R_{inj}}^{R_{max}} dR_b (4\pi/3) R_b^3 Q(R_b) F(R_b). \qquad [9]$$

The specific choices in parameters made here yield the results shown in figure 2 of the main text, which lead to excellent agreement when comparing to supersaturation data at high wind speed.

5. Air entrainment in the full spectral model

The air entrainment model has been described in previous work (4, 13, 24, 34, 35). We summarize the main elements. (34) showed that the bubble flux entrained is provided by the third moment of $\Lambda(c)$ for a given sea state (wave spectra) and wind speed. The volume of air entrained is constrained by laboratory experiments and direct numerical simulations (15, 16, 27) and the rate of entrainment of air per unit area of ocean surface, V_A (dimensions of a volume per area per time),

$$V_A = \int B s(k)^{3/2} \frac{c^3}{g} \Lambda(c)\, \mathrm{d}c, \qquad [10]$$

where $s(k)$ is the wave slope, and $B \approx 0.12$ is a constant (4, 16). The wave slope within a complex wave field can be estimated from the wave spectrum $\phi(k)$, with k the wave number, linked to the wave speed by $c = \sqrt{g/k}$, and (36, 37) we consider $b/hk = s(k)^{3/2} = A_T(\sqrt{k^3\phi(k)} - \sqrt{B_T})^{3/2}$ using the same constants A_T and B_T as for energy dissipation (36, 38). The volume flux V_A entrained by breaking waves is the volumetric analog of the whitecap coverage (13, 34). The volume flux is by definition related to the bubble size distribution under a breaking wave $q(R_b)$: $V_A = \int dR_b(4\pi R_b^3)/3Q(R_b)$. Entrainment depth in the full spectral model is taken as $z_0 \approx H_s/2$ with H_s the significant wave height, as described in (13, 34). These assumptions lead to the air entrainment scaling in the field $V_A \propto u_*^{5/3}\sqrt{gH_s}^{4/3}$, used for the wind-wave-bubble model eqs. 9 and 10 in the main text.

As described in (4), we evaluate the sea state dependent sea spray generation function by using a global spectral wave model (WAVEWATCH III, WW3, (39)), able to simulate the growth and propagation of wave energy of the wave spectrum $\phi(k)$, considering wind forcing from an atmospheric product. We utilize the breaking statistics model from (37) that relates the wave spectrum $\phi(k)$ to the breaking distribution $\Lambda(c)$. We perform global WW3 simulations forced by the Japanese Meteorological Society Reanalysis product (JRA55-do v1.5), which provides ten-meter wind vectors (U_{10}) at approximately half-degree resolution every three hours (1, 2).

The formulation explicitly considers the sea state effects through the $\Lambda(c)$ distribution and the wave spectrum $\phi(k)$, controlling the number flux of bubbles $Q(R_b)$ and the air entrainment V_A. Physico-chemical variables, such as temperature and salinity control the gas solubility and diffusivity, modulate the liquid viscosity; and are evaluated as in (4), see also table S2 for references to how the different variables are calculated. We remind the reader that the results for CO_2 are fully compatible with existing eddy covariance data, as described in detail in (4) where the formulation was validated against extensive data sets for CO_2 considering the surface (non-breaking) and bubble symmetric terms.

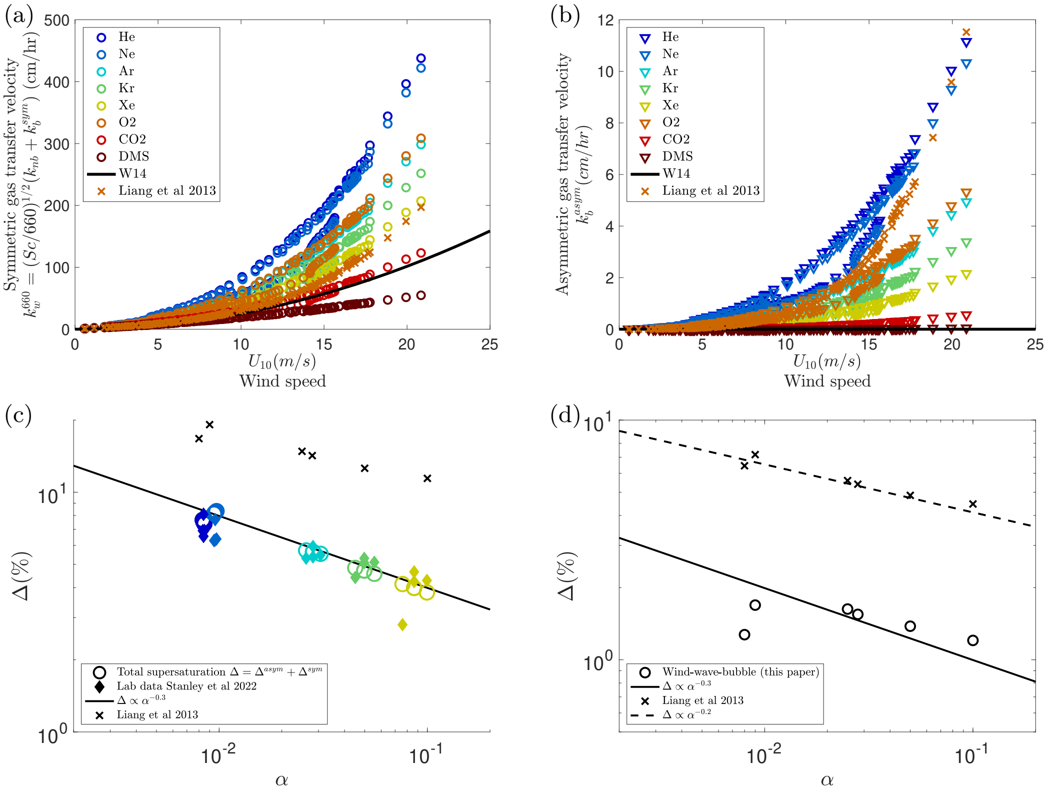

Fig. S4. (a,b) Gas transfer velocity (symmetric, a; and asymmetric contributions, b) as a function of gas solubility and wind speed for a wide range of gases, compared to Liang et al,. (2013) (40) for O_2. (c) Associated bubble induced supersaturation calculated in the high wind speed regime from SUSTAIN (at 50 m s^{-1}). (d) Associated supersaturation comparing our new wind-wave-bubble formulation to Liang et al,. (2013) at 20 m s^{-1} (crosses).

6. Comparison to Liang et al,. (2013) (40)

Figure S5 shows a comparison of our wind-wave-bubble formulation to the parameterization proposed by Liang et al,. (2013) (40), showing figures similar to those in the main text. Both Stanley et al., (2009) and Liang et al,. (2013) yield a smaller symmetric gas transfer contribution (with no solubility dependency) but a stronger asymmetric contribution than ours (figs. S4a-b). The associated supersaturation (fig. S4c) is significantly higher using Liang et al,. (2013) (crosses) (and even larger for Stanley et al,. 2009), due to a very strong dependence in wind speed, that leads to unrealistic values in the high wind speed conditions (50 m s^{-1}) plotted here. A weaker dependence in solubility α than our work and the laboratory data is observed.

The supersaturation at 20 m s^{-1} is also shown (fig. S4d) and again, a weaker dependence in solubility α is observed for Liang et al,. (2013) compared to ours, and the magnitude is significantly higher. Results from Stanley et al,. 2009 would be even higher.

7. Momentum flux (drag coefficient) formulation

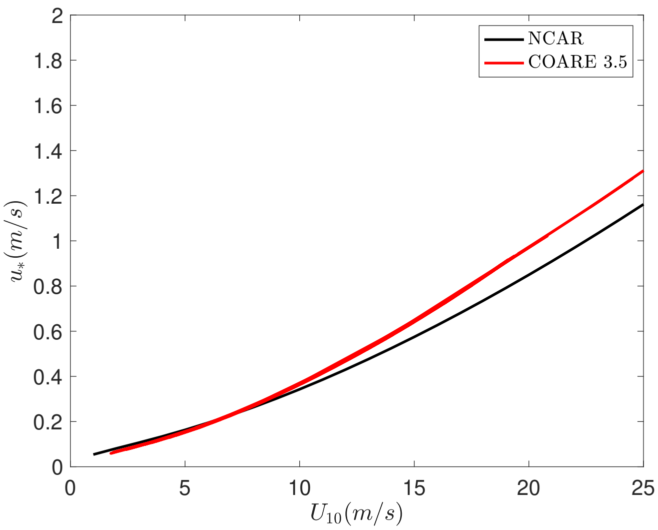

Fig. S5. Comparison between the NCAR and COARE momentum flux (drag coefficient) formulations in terms of the wind friction velocity as a function of the neutral wind speed at 10 m. Details of each formulation are provided in (5, 6).

We show in Figure S5 the COARE and NCAR momentum flux (drag coefficient) formulations, presented in terms of the wind friction velocity as a function of wind speed (neutral wind speed at 10 m). The corresponding formula for the drag coefficient is provided in (5, 6).

8. Wind-wave-bubble based on wind speed only formulation

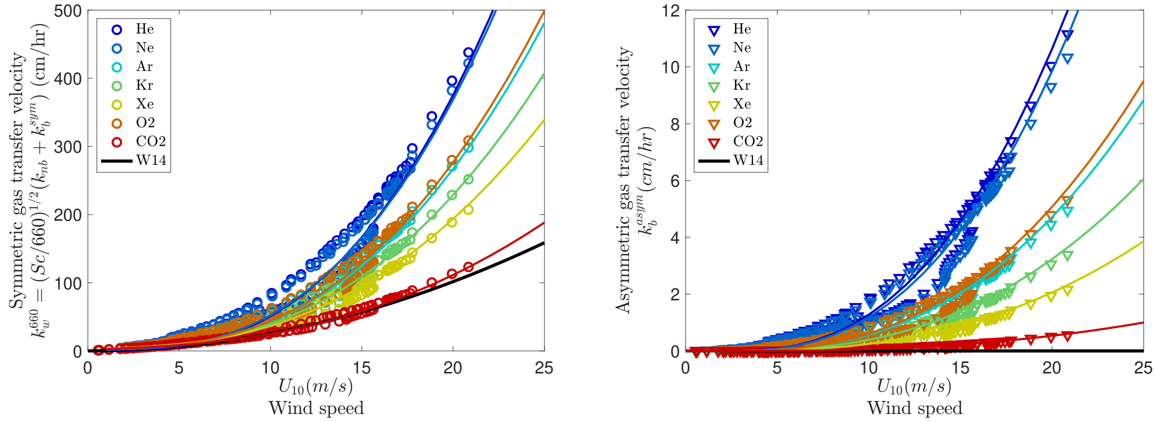

Fig. S6. Comparison between the new wind-wave-bubble formulation (symbols) already shown in Figures 3 and S4; and the wind-wave-bubble based on wind speed only (see equations in table 1) with solid lines.

We show in Figure S6 the wind-wave-bubble based on wind speed only (see equations in table 1) compared to the wind-wave-bubble formulation used in the paper and shown in Figures 3 and S4. The wind-only one accounting for bubble effects provides similar mean values at a given wind speed without the wave-induced variability.

9. Sensitivity tests to z_0 and R_{inj} for the laboratory conditions from Stanley et al 2022 (41)

In this section, we illustrate the sensitivity of the bubble theory to the injection depth z_0 in figure S7 and cut-off size R_{inj} in figure S8. The analysis is done with otherwise the same parameters used for figure 2 in the main paper, i.e. high wind speed conditions and the bubble size distribution, rise velocity and individual transfer rate (all given in section 4 of the SI).

We consider four values of z_0, 10cm, 25cm, 50cm (the one used in figure 2) and 1m to illustrate the sensitivity of the various terms. We note that the choice of 0.5m is the most physical given the laboratory conditions (41). An injection depth of 10cm is too shallow while 1m would be too large (almost all the water depth) in the specific laboratory setup, so these calculations have to be taken as an illustration of the sensitivity of the equations. The choice made in the main paper ($z_0 = 0.5$m) leads to the best comparison with the supersaturation laboratory data and guides the derivation of the simple wind-wave-bubble formulation (eq. 9 and 10) as it controls the scaling in solubility and diffusivity.

The exchanged volume (calculated from eq. 2 in the SI) is shown (fig. S7a) demonstrating very large sensitivity to z_0 at low solubility, and weaker at higher; so that the exchanged volume scaling with α changes when z_0 is changed. The exchanged volume increases with increasing injection depth as the residence time of the bubble is longer. The symmetric transfer velocity (fig. 7b) calculated from eq. 7 in this document $k_b^{sym} = V_{exch}/\alpha$ is as a consequence also very sensitive to the injection depth.

The overpressure (calculated from eq. 5 in the SI) is similarly a very strong function of the injection depth, which is intuitive since as z_0 increases, the bubbles are subject to higher pressure. Again, the scaling with α is changed when z_0 is changed with a weaker dependency at low z_0. The asymmetric gas transfer velocity (fig. S7d), calculated from eq. 8 in the SM is mostly affected in its magnitude (as it combines the two terms coming from the larger exchanged and smaller injected bubbles).

Finally, the resulting supersaturation for these four injection depth is shown in fig. S7e,f. Total supersaturation is shown in S7e, with higher injection depth leading to higher values of supersaturation, and a weaker dependency in solubility. This can be understood by the fact that the deeper the bubbles are injected, the more compressed the large bubbles will be leading to higher supersaturation regardless of the solubility. We observe that the choice of $z_0 = 0.5$m already shown in figure 2 of the main paper yields excellent agreement with the laboratory data while smaller injection depth fail at high solubility and higher injection depth leads to systematically too high supersaturation. Figure S7f shows the details of the two contribution to the supersaturation (through the exchanged term, labeled asymmetric and the injected term, labeled symmetric, see eqs in methods) showing that for shallow injection, the injected term dominates while for deeper injection, both are important, as discussed in the main paper.

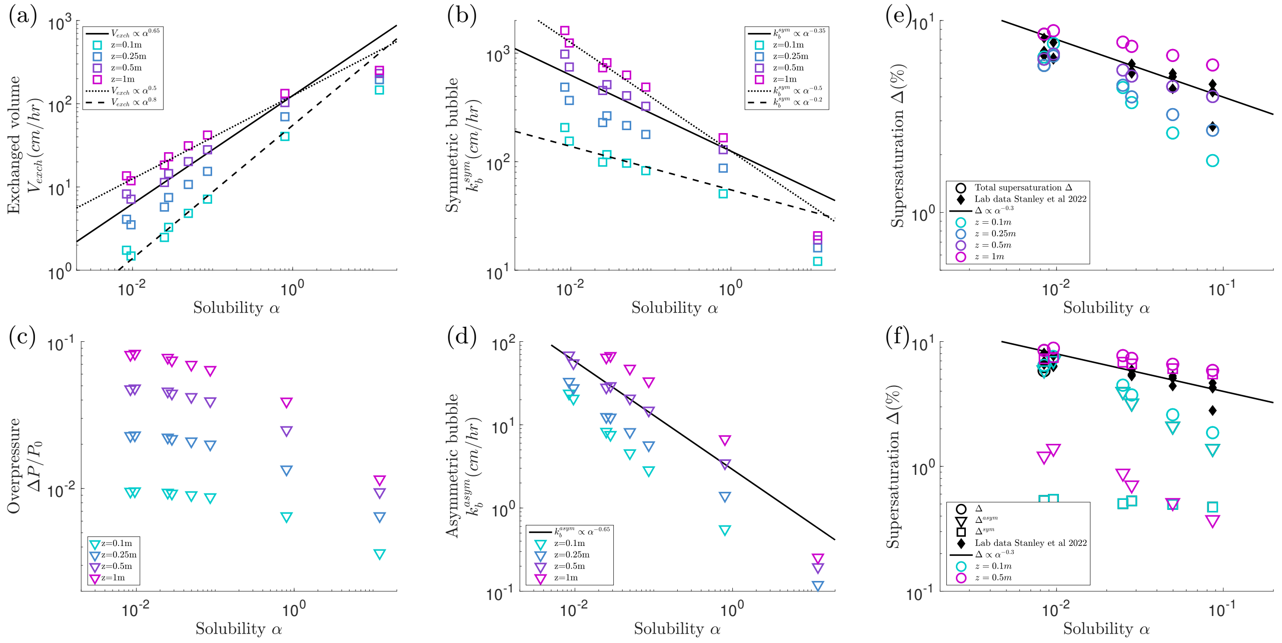

Fig. S7. Illustration of the sensitivity to the bubble theory to the injection depth z_0. The exchanged volume (a) is sensitive to z_0 in magnitude and dependence in solubility magnitude and dependence in solubility. As a consequence, the symmetric bubble gas transfer velocity (b) is highly sensitive to the choice of injection depth. Similarly, the overpressure (c) is highly sensitive to z_0 in magnitude and dependence in solubility magnitude and dependence in solubility and as a consequence, the asymmetric bubble gas transfer velocity (d) is also sensitive to z_0. And as a consequence, the supersaturation (e,f) does also vary with changes in z_0. The results for $z_0 = 0.5$m correspond to the ones shown in the main paper, leading to the best agreement with the supersaturation data observed in the laboratory and are the one used to derive the simple wind-wave-bubble model.

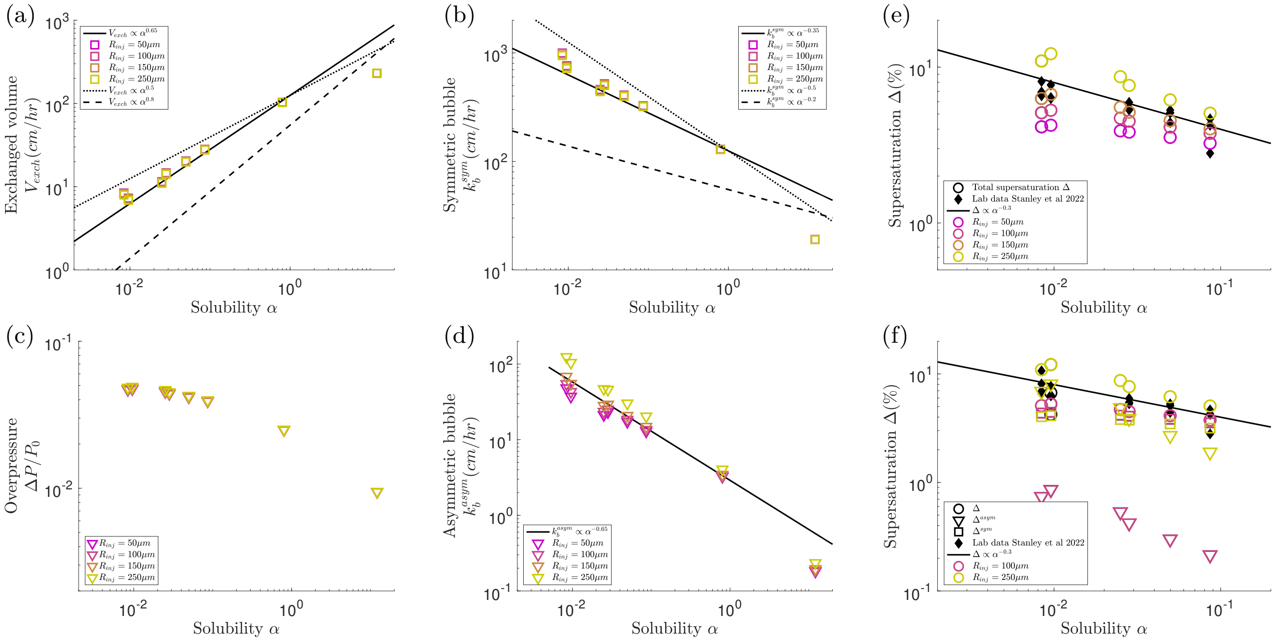

Fig. S8. Illustration of the sensitivity to the bubble theory to the cut-off size R_{inj} between small (injected) and large (exchanged) bubbles (for constant $z_0 = 0.5$m). The exchanged volume (a) is not sensitive to the changes in R_{inj}, so that the symmetric bubble gas transfer velocity is also not sensitive (b). The overpressure (c) is similarly not sensitive to R_{inj}. However, since V_{inj} is increased when R_{inj} is increased, the asymmetric bubble contribution (d) is sensitive to R_{inj}. And as a consequence, the supersaturation (e,f) does also vary with changes in R_{inj}. The results for $R_{inj} = 150\mu$m correspond to the ones shown in the main paper, leading to the best agreement with the supersaturation data observed in the laboratory and are the one used to derive the simple wind-wave-bubble model.

Similar analysis can be performed on the cut-off size R_{inj} and is shown in figure S8. For a fixed z_0 (taken as 0.5m as in the comparison to the laboratory in figure 2 main manuscript), variations of R_{inj} within the physical range (from 50 to 250 microns) has very little influence on the exchanged volume (fig. S8a) and symmetric gas transfer velocity (fig. S8b) and overpressure (fig. S8c). It however influences directly V_{inj} and therefore the asymmetric transfer velocity (fig. S8d) and the resulting supersaturation (shown in fig. S8e,f). Again the choice made in the main paper ($z_0 = 0.5$m, $R_{inj} = 150\mu$m) leads to the best comparison with the supersaturation laboratory data and guides the derivation of the simple wind-wave-bubble formulation (eq. 9 and 10) as it controls the scaling in solubility and diffusivity. Smaller or larger cut offs R_{inj} lead to either too weak or too high supersaturation at low solubility.

While developing the theoretical model, we also tested the sensitivity to all other parameters within ranges suggested by available knowledge on bubble physics under breaking waves (24): rise velocity formulations and individual bubble gas transfer formulations, accounting for turbulence effect; minimum or maximum bubble size, shape of the bubble size distribution, etc. Similar tests were presented in the original paper by Keeling 1993 (22). We also note that the various parameters are also expected to be all interlinked in the actual ocean (we would expect the injection depth to scale with the wave height and wind speed, which would also influence the cut-off size for example) and the final transfer velocity and supersaturation (and how they depend on solubility and diffusivity) are non-trivial functions of these dependencies. In the present study, we have used the best of our knowledge on the various bubble quantities and demonstrated that it allows to reproduce several laboratory data (as shown in figure 2 main paper and again in the SI), and inform a simple formulation (and associated uncertainties) which is then implemented in the large scale ocean model.

References

1. S Kobayashi, et al., The jra-55 reanalysis: General specifications and basic characteristics. *J. Meteorol. Soc. Jpn. Ser. II* **93**, 5–48 (2015).
2. H Tsujino, et al., Jra-55 based surface dataset for driving ocean–sea-ice models (jra55-do). *Ocean. Model.* **130**, 79–139 (2018).
3. BG Reichl, L Deike, Contribution of sea-state dependent bubbles to air-sea carbon dioxide fluxes. *Geophys. Res. Lett.*, e2020GL087267 (2020).
4. X Zhou, BG Reichl, L Romero, L Deike, A sea state dependent gas transfer velocity for co2 unifying theory, model, and field data. *Earth Space Sci.* **10**, e2023EA003237 (2023).
5. WG Large, SG Yeager, Diurnal to decadal global forcing for ocean and sea-ice models: The data sets and flux climatologies (2004).
6. J Edson, et al., On the exchange of momentum over the open ocean. *J. Phys. Oceanogr.* **43**, 1589–1610 (2013).
7. R Weiss, Carbon dioxide in water and seawater: the solubility of a non-ideal gas. *Mar. chemistry* **2**, 203–215 (1974).

8. R Wanninkhof, W Asher, D Ho, C Sweeney, W McGillis, Advances in quantifying air-sea gas exchange and environmental forcing. *Annu. Rev. Mar. Sci.* (2009).
9. KG Nayar, MH Sharqawy, LD Banchik, , et al., Thermophysical properties of seawater: A review and new correlations that include pressure dependence. *Desalination* **390**, 1–24 (2016).
10. RH Stanley, WJ Jenkins, DE Lott III, SC Doney, Noble gas constraints on air-sea gas exchange and bubble fluxes. *J. Geophys. Res. Ocean.* **114** (2009).
11. RC Hamme, DP Nicholson, WJ Jenkins, SR Emerson, Using noble gases to assess the ocean's carbon pumps. *Annu. Rev. Mar. Sci.* **11**, 75–103 (2019).
12. RH Stanley, WJ Jenkins, DE Lott III, SC Doney, Noble gas constraints on air-sea gas exchange and bubble fluxes. *J. Geophys. Res. Ocean.* **114** (2009).
13. L Deike, WK Melville, Gas transfer by breaking waves. *Geophys. Res. Lett.* **45**, 10–482 (2018).
14. E Lamarre, W Melville, Air entrainment and dissipation in breaking waves. *Nature* **351**, 469–472 (1991).
15. GB Deane, MD Stokes, Scale dependence of bubble creation mechanisms in breaking waves. *Nature* **418**, 839–844 (2002).
16. L Deike, W Melville, S Popinet, Air entrainment and bubble statistics in breaking waves. *J. Fluid Mech.* **801**, 91–129 (2016).
17. AH Callaghan, GB Deane, DM Stokes, Two regimes of laboratory whitecap foam decay: bubble-plume controlled and surfactant stabilized. *J. Phys. Oceanogr.* (2013).
18. DJ Ruth, et al., Three-dimensional measurements of air entrainment and enhanced bubble transport during wave breaking. *Geophys. Res. Lett.* **49**, e2022GL099436 (2022).
19. PA Bowyer, Video measurements of near-surface bubble spectra. *J. Geophys. Res. Ocean.* **106**, 14179–14190 (2001).
20. L Lenain, W Melville, Evidence of sea-state dependence of aerosol concentration in the marine atmospheric boundary layer. *J. Phys. Oceanogr.* **47**, 69–84 (2017).
21. DK Woolf, S Thorpe, Bubbles and the air-sea exchange of gases in near-saturation conditions. *J. Mar. Res.* **49**, 435–466 (1991).
22. RF Keeling, On the role of large bubbles in air-sea gas exchange and supersaturation in the ocean. *J. Mar. Res.* **51**, 237–271 (1993).
23. S Thorpe, On the clouds of bubbles formed by breaking wind-waves in deep water, and their role in air–sea gas transfer. *Philos. Transactions Royal Soc. Lond. A: Math. Phys. Eng. Sci.* **304**, 155–210 (1982).
24. L Deike, Mass transfer at the ocean–atmosphere interface: The role of wave breaking, droplets, and bubbles. *Annu. Rev. Fluid Mech.* **54** (2022).
25. G Rojas, M Loewen, Fiber-optic probe measurements of void fraction and bubble size distributions beneath breaking waves. *Exp. Fluids* **43**, 895–906 (2007).
26. CE Blenkinsopp, JR Chaplin, Bubble size measurements in breaking waves using optical fiber phase detection probes. *Ocean. Eng. IEEE J.* **35**, 388–401 (2010).
27. W Mostert, S Popinet, L Deike, High-resolution direct simulation of deep water breaking waves: transition to turbulence, bubbles and droplets production. *J. Fluid Mech.* **942**, A27 (2022).
28. A Rivière, W Mostert, S Perrard, L Deike, Sub-hinze scale bubble production in turbulent bubble break-up. *J. Fluid Mech.* **917** (2021).
29. A Rivière, DJ Ruth, W Mostert, L Deike, S Perrard, Capillary driven fragmentation of large gas bubbles in turbulence. *Phys. Rev. Fluids* **7**, 083602 (2022).
30. JO Hinze, Fundamentals of the hydrodynamic mechanism of splitting in dispersion processes. *AIChE J.* **1**, 289–295 (1955).
31. C Garrett, M Li, D Farmer, The connection between bubble size spectra and energy dissipation rates in the upper ocean. *J. Phys. Ocean.* **30**, 2163–2171 (2000).
32. H Czerski, et al., Ocean bubbles under high wind conditions–part 1: Bubble distribution and development. *Ocean. Sci.* **18**, 565–586 (2022).
33. H Czerski, et al., Ocean bubbles under high wind conditions–part 2: Bubble size distributions and implications for models of bubble dynamics. *Ocean. Sci.* **18**, 587–608 (2022).
34. L Deike, L Lenain, WK Melville, Air entrainment by breaking waves. *Geophys. Res. Lett.* **44**, 3779–3787 (2017).
35. L Deike, B Reichl, F Paulot, A mechanistic sea spray generation function based on the sea state and the physics of bubble bursting. *AGU Adv.* **3**, e2022AV000750 (2022).
36. L Romero, WK Melville, JM Kleiss, Spectral energy dissipation due to surface wave breaking. *J. Phys. Ocean.* **42**, 1421–1441 (2012).
37. L Romero, Distribution of surface wave breaking fronts. *Geophys. Res. Lett.* **46**, 10463–10474 (2019).
38. DA Drazen, WK Melville, L Lenain, Inertial scaling of dissipation in unsteady breaking waves. *J. Fluid Mech.* **611**, 307–332 (2008).
39. WW3DG, User manual and system documentation of WAVEWATCH III version 5.16., (NOAA/NWS/NCEP/MMAB, College Park, MD, USA), Technical report (2016).
40. JH Liang, et al., Parameterizing bubble-mediated air-sea gas exchange and its effect on ocean ventilation. *Glob. Biogeochem. Cycles* **27**, 894–905 (2013).
41. RH Stanley, et al., Gas fluxes and steady state saturation anomalies at very high wind speeds. *J. Geophys. Res. Ocean.* **127**, e2021JC018387 (2022).
